# Predicting genomic selection efficiency to optimize calibration set and to assess prediction accuracy in highly structured populations

**DOI:** 10.1007/s00122-017-2956-7

**Published:** 2017-08-09

**Authors:** R. Rincent, A. Charcosset, L. Moreau

**Affiliations:** 1 INRA, UMR 1095 Génétique, Diversité et Ecophysiologie des Céréales, 5 chemin de Beaulieu, 63100 Clermont-Ferrand, France; 2 Université Blaise Pascal, UMR 1095 Génétique, Diversité et Ecophysiologie des Céréales, 63178 Aubière Cedex, France; 3 UMR de Génétique Végétale, INRA – Université Paris-Sud – CNRS, 91190 Gif-Sur-Yvette, France

## Abstract

*****Key message***:**

**We propose a criterion to predict genomic selection efficiency for structured populations. This criterion is useful to define optimal calibration set and to estimate prediction reliability for multiparental populations.**

**Abstract:**

Genomic selection refers to the use of genotypic information for predicting the performance of selection candidates. It has been shown that prediction accuracy depends on various parameters including the composition of the calibration set (CS). Assessing the level of accuracy of a given prediction scenario is of highest importance because it can be used to optimize CS sampling before collecting phenotypes, and once the breeding values are predicted it informs the breeders about the reliability of these predictions. Different criteria were proposed to optimize CS sampling in highly diverse panels, which can be useful to screen collections of genotypes. But plant breeders often work on structured material such as biparental or multiparental populations, for which these criteria are less adapted. We derived from the generalized coefficient of determination (CD) theory different criteria to optimize CS sampling and to assess the reliability associated to predictions in structured populations. These criteria were evaluated on two nested association mapping (NAM) populations and two highly diverse panels of maize. They were efficient to sample optimized CS in most situations. They could also estimate at least partly the reliability associated to predictions between NAM families, but they could not estimate differences in the reliability associated to the predictions of NAM families using the highly diverse panels as calibration sets. We illustrated that the CD criteria could be adapted to various prediction scenarios including inter and intra-family predictions, resulting in higher prediction accuracies.

**Electronic supplementary material:**

The online version of this article (doi:10.1007/s00122-017-2956-7) contains supplementary material, which is available to authorized users.

## Introduction

Classical plant breeding programs rely on the phenotyping of progenies in field trial networks to identify superior individuals. The number of individuals which can be evaluated is limited by high phenotyping costs and time needed to perform relevant field evaluation. This reduced number of selection candidates is a major limit to genetic progress. Genomic selection (GS) allows predicting the performance of unphenotyped individuals, which makes it possible to increase the size of the candidate set (Meuwissen et al. 2001). GS prediction equations are calibrated using phenotypes and genotypes of the reference individuals composing the calibration set. The equations can then be used to predict the genomic estimated breeding values (GEBV) of selection candidates, as long as their genotype is available. As genotyping tools such as SNP-arrays are now available and affordable for many species, GS is becoming a reference tool for breeders, and greatly complements marker assisted selection tools based on QTL detection.

The optimal use of GS in plant breeding depends on the species and is influenced by many factors (length of the selection cycle, importance of genotype x environment interactions, selection based on hybrids or on inbred lines, technical tools available…). There are nevertheless two common major opportunities brought by GS in breeding: (i) the screening of highly diverse material in pre-breeding steps (Crossa et al. 2016; Yu et al. 2016), and (ii) the prediction of performance of selection candidates in the breeding programs. In (i) and (ii), the phenotyping costs saved by GS can be spend for a more intense phenotyping of the calibration set: phenotyping for traits difficult to measure, phenotyping in more environments, or phenotyping of crosses with more testers (in the case of hybrid breeding).

It has been shown, that the efficiency of GS is affected by various factors linked to the predicted trait [genetic architecture, heritability (Heffner et al. 2009; Hayes et al. 2009; Jannink 2010)], to the population under study [linkage disequilibrium (Heffner et al. 2010; Albrecht et al. 2011), structure and relatedness (Wientjes et al. 2013; Albrecht et al. 2014; Lehermeier et al. 2014)], to the statistical model used (Heslot et al. 2012), to the genotypic information available (Chen and Sullivan 2003; Poland and Rife 2012), and to the calibration set (Habier et al. 2010; Albrecht et al. 2011; Pszczola et al. 2012). Among these, the composition of the calibration set highly influences prediction accuracy, and thus genetic progress. It was shown for instance that prediction accuracy increases with the level of relatedness between the calibration set and the test set (Habier et al. 2010; Albrecht et al. 2011; Pszczola et al. 2012).

Different criteria (prediction error variance PEV, or the coefficient of determination) are available to estimate the expected accuracy of G-BLUP, one reference GS model, for given calibration and test sets. He et al. (2016) used the expected PEV of individual predictions in a commercial wheat program and showed its efficiency to identify individuals outside the calibration space and, therefore, poorly predicted. The interest lies very often in the estimation of contrasts between individuals, for instance the identification of superior segregating individuals compared to the family mean or compared to checks. The precision of any contrast of genetic values can be estimated with the generalized coefficient of determination (further noted CD), defined as the squared correlation between the true and the predicted contrast of genetic values (Laloë 1993). The CD is the expected reliability of the contrast. This criterion was first introduced in the context of GS by Maenhout et al. (2010) to select optimal subsets of phenotypic data and by Rincent et al. (2012) to optimize the composition of the calibration set using genotyping data only. The information brought by the CD can be very useful to breeders at different steps of the selection program. The CD can first be used to sample an optimal calibration set to be phenotyped (Rincent et al. 2012). Once the calibration set has been sampled, a second potential use of the CD is to evaluate a priori the reliability of the predictions. It is indeed clear that the selections operated by breeders in the context of GS should be made by considering both performance predictions and the reliability of these predictions. This is similar to classical breeding, in which breeders select individuals by considering both adjusted means and the accuracies of the trials.

Rincent et al. (2012) derived from the CD a criterion to maximize prediction accuracy in highly diverse panels (CDmean). CDmean was successfully tested in different species such as maize, palm trees, wheat and peas in populations of various levels of relatedness (Rincent et al. 2012; Rutkoski et al. 2015; Cros et al. 2015; Tayeh et al. 2015). Isidro et al. (2015) showed that CDmean performed less efficiently in structured populations including subspecies, or when trait architecture potentially involved major effect genes. Note, however, that the genotypic information of the test set was not taken into account in this latter study, which might partly explain the poor efficiency of the CDmean that was observed. It is nevertheless true that the CDmean proposed by Rincent et al. (2012) doesn’t take into account strong population structures. Another criterion is therefore needed to optimize calibration set in materials in such situations, which are common in plant breeding programs. Indeed, when panels of inbred lines or previously existing biparental populations are used to predict performances of individuals from new biparental populations, population structure differs in the calibration set and the prediction set. More work needs to be done to evaluate in this case the efficiency of criteria based on CD to predict in advance the efficiency of GS and to optimize the calibration set according to the breeding population targeted. We propose and evaluate in this study a new criterion (CDpop), also derived from the generalized CD, but based on contrasts adapted to structured material. The Dent and Flint nested association mapping (NAM) populations presented in Bauer et al. (2013), and Lehermeier et al. (2014) is an excellent material to evaluate the efficiency of CDpop, because it is clearly structured in biparental families and presents variability in both within and between family relatedness. CDpop was tested for the two objectives presented above: (i) predict the efficiency of a given calibration set to predict a given family, and (ii) optimize the composition of the calibration set to predict a given population. In (i) the breeder wants to evaluate how much he should trust his predictions, and in (ii) the breeder wants to sample an optimal calibration set prior to phenotyping. The efficiency of CDpop for (i) and (ii) was also evaluated when highly diverse panels such as those presented in Rincent et al. (2012) and Rincent et al. (2014a) are used to predict NAM families.

## Materials and methods

### Plant material and phenotypic analysis

#### Diversity panels

The two Dent and Flint panels of the “CornFed” program (CF-Dent and CF-Flint) were developed to analyze diversity and linkage disequilibrium in two heterotic groups of main interest for maize hybrid breeding in Northern Europe (Rincent et al. 2012, 2014a, b). Both panels are composed of 300 inbred lines aiming at best representing the diversity of these heterotic groups and different generations of genetic materials. These include the first commercially used inbred lines created from open pollinated varieties (OPVs), and more recent inbred lines developed by public institutes or, in the case of the CF-Dent panel, private companies. For phenotypic evaluation, the inbred lines of a given panel were crossed with a tester of the other pool (Dent inbred lines crossed to UH007, and Flint inbred lines crossed to F353). All hybrids were evaluated for male flowering time (anthesis date, AD, in days after sowing), plant height (PH, cm), and dry matter yield (DMY, Mg/ha). Two separate experiments were conducted for the Dent and Flint hybrids, with five locations for each panel in 2010, and six (CF-Dent) and five (CF-Flint) locations in 2011. In this study we used the least-square means of the hybrids as computed by Rincent et al. (2014b).

#### NAM populations

The two NAM designs are described by Bauer et al. (2013). In short, the Dent and Flint populations were, respectively, composed of 10 and 11 doubled haploid (DH) families, derived from the cross of, respectively, 10 and 11 diverse founder inbred lines with a common central inbred line: F353 for the Dent and UH007 for the Flint. F353 and UH007 represent European inbred lines created by public institutes in their respective heterotic groups. The parental inbred lines were chosen to cover the diversity available within the two heterotic groups with a combination of ancestral and more recent material. All parental inbred lines are included in the CF-Dent or CF-Flint panels. From each cross, doubled haploid (DH) lines were generated, resulting in 919 lines for the Dent and 1009 for the Flint (Bauer et al. 2013). For phenotypic evaluation, the segregating DH lines of a given heterotic group were crossed with the central inbred line of the other heterotic group (corresponding to the same testers used for the CF-Dent and CF-Flint panels). A total of 841 hybrids were produced for the Dent heterotic group and 811 for the Flint heterotic group (Lehermeier et al. 2014). The number of Dent DH lines for which testcrossed progenies were phenotyped per family was 84 on average and varied between 53 and 104, depending on the family. For the Flint heterotic group, the number of DH lines per family that were phenotyped for testcross values ranged from 17 to 133 with an average of 73. Hybrids were evaluated in 2011 in four (Dent) and six (Flint) European locations for the same traits than the CF-Dent and CF-Flint panels. Field trial design is described in Lehermeier et al. (2014). In their study individual field plot measures were analyzed to compute for each hybrid the adjusted means over the different trials. We used the same adjusted means in the present study. The Flint DH family resulting from the cross of EP44 and UH007 was not used due to small population size.

### Genotypic data and relatedness

#### Genotypic data

The 1894 NAM DH lines (corresponding to the 10 Dent and 10 Flint families), the 22 parental inbred lines and the two diversity panels were genotyped with the Illumina MaizeSNP50 BeadChip containing 56,110 SNPs (Ganal et al. 2011). For the diversity panels we used the data filtered and imputed as in Rincent et al. (2014a, b). For the NAM populations, markers with a call frequency <0.9, a GenTrain Score <0.7, or >10% missing values were discarded as described by Lehermeier et al. (2014).

We only considered PANZEA SNP in our study to avoid ascertainment bias in kinship estimation (Ganal et al. 2011). Among these markers, we only kept those which passed the quality filters in both the NAM populations and the diversity panels and which had a minor allele frequency (MAF) above 0.01 in the diversity panels, which resulted in 27,169 markers in the Dent NAM families and in the CF-Dent panel, and 26,920 markers in the Flint NAM families and in the CF-Flint panel. In the NAM families missing values were imputed as the mean allelic frequency in the corresponding NAM family.

Individuals without phenotypes, or with >10% missing values were discarded. As a result, the NAM populations comprised 841 and 794 lines in the Dent and Flint NAM populations. The diversity panels were composed of 281 and 275 individuals for CF-Dent and CF-Flint panels, respectively. Genotypic data of each heterotic group (Dent and Flint) were organized as *G* matrices with *N* rows and *L* columns, *N* and *L* being the number of genotypes and of SNP loci, respectively. Genotype of individual *i* at marker *l* (G_*i,l*_) was coded as 1, 0.5, or 0 for homozygote for an arbitrarily chosen allele, heterozygote, and the other homozygote, respectively.

#### Kinship estimation

Kinship within and between NAM families and panel for each heterotic group (Dent and Flint) was estimated using the PANZEA SNP following VanRaden (2008):

$$K_{i,j} = \mathop \sum \limits_{l = 1}^{L} \frac{{\left( {G_{i,l} - p_{l} } \right)\left( {G_{j,l} - p_{l} } \right)}}{D},$$

with $D = \mathop \sum \nolimits_{l = 1}^{L} p_{l} \times \left( {1 - p_{l} } \right),$ $p_{l}$ being the allelic frequency of the reference allele in the corresponding diversity panel, L the number of markers.

### Genome-based prediction model and generalized coefficient of determination

#### Genome-based prediction model

The genomic predictions were based on the G-BLUP model, using the following mixed model:

$$\varvec{y} = \varvec{X\beta } + \varvec{Zu} + \varvec{e, }$$

where ***y*** is a vector of phenotypes consisting of adjusted means, **β** is a vector of fixed effects (in our case only the intercept), ***u*** is a vector of random genetic values, ***e*** is the vector of residuals. ***X*** and ***Z*** are design matrices.

***u*** was assumed to follow a Gaussian distribution: $\varvec{u }\sim \varvec{ N}\left( {\vec{\bf 0},\varvec{K}\sigma_{g}^{2} } \right)$, where ***K*** is the genomic relationship matrix estimated as above, and $\sigma_{g}^{2}$ is the additive genetic variance. The residuals ***e*** are assumed to follow a Gaussian distribution: $\varvec{ e }\sim \varvec{N}\left( {\vec{\bf 0},\varvec{I}\sigma_{e}^{2} } \right)$, where ***I*** is the identity matrix. The prediction of ***u*** is obtained by solving Henderson’s equations (Henderson 1984):

$$\left[ {\begin{array}{*{20}c} {\varvec{X^{\prime}X}} & {\varvec{X} '\varvec{Z}} \\ {\varvec{Z} '\varvec{X}} & {\varvec{Z} '\varvec{Z} + \lambda \varvec{K}^{ - 1} } \\ \end{array} } \right]\left[ {\begin{array}{*{20}c} {\hat{\varvec{\beta }}} \\ {\hat{\varvec{u}}} \\ \end{array} } \right] = \left[ {\begin{array}{*{20}c} {\varvec{X} '\varvec{y}} \\ {\varvec{Z} '\varvec{y}} \\ \end{array} } \right] ,$$

where $\lambda = \frac{{\sigma_{e}^{2} }}{{\sigma_{g}^{2} }}$ is the ratio between the residual and the additive variances. In practice $\lambda$ can be set using an estimation of the heritability of the trait or the restricted maximum likelihood (REML) estimates of $\sigma_{g}^{2}$ and $\sigma_{e}^{2}$ using the phenotypic data of the calibration set.

### Generalized coefficient of determination (CD)

Before collecting phenotypes it is possible to estimate the prediction reliability of different contrasts using the generalized CD (Laloë 1993). It is defined as the squared correlation between the true and the predicted contrast of genetic values. It is equivalent to the expected reliability of the contrast:

$$\varvec{CD}\left( \varvec{c} \right) = {\text{diag}}\left[ {\frac{{\varvec{c}^{\prime } \left( {\varvec{K} - \lambda \left( {\varvec{Z^{\prime}MZ} + \lambda \varvec{K}^{ - 1} } \right)^{ - 1} } \right)\varvec{c}}}{{\varvec{c}^{\prime } \varvec{Kc}}}} \right],$$

where **c** is a contrast, i.e., $1^{\prime } \varvec{c} = 0$. **M** is an orthogonal projector on the subspace spanned by the columns of ***X***: $\varvec{M} = \varvec{I} - \varvec{X}\left( {\varvec{X}^{\prime } \varvec{X}} \right)^{ - } \varvec{X}^{\prime }$ and $\left( {\varvec{X}^{\prime } \varvec{X}} \right)^{ - }$ is a generalized inverse of ***X’X*** (Laloë 1993). The CD takes values between 0 and 1, a CD close to 0 meaning that the prediction of the contrast is not reliable, whereas CD close to 1 meaning that the prediction is highly reliable, i.e., the predicted and the true genetic values are expected to be strongly correlated. The CD is related to the prediction error variance (PEV) of the contrast as expressed in Laloë 1993:

$\varvec{PEV}\left( \varvec{c} \right) = diag\left[ {\frac{{\varvec{c}^{\prime } \left( {\varvec{Z}^{\prime } \varvec{MZ} + \lambda \varvec{K}^{ - 1} } \right)^{ - 1} \varvec{c}}}{{\varvec{c}^{\prime } \varvec{c}}}} \right]\sigma_{e}^{2}$. Criteria related to PEV were also evaluated to optimize calibration set sampling (Rincent et al. 2012; Akdemir et al. 2015). Note that Akdemir et al. (2015) considered individual PEV and not PEV of contrasts between individuals.

Depending on the objective, one can consider different contrasts **c**. In this study we are interested mostly in making predictions within each NAM family (using phenotypes collected in the panels, or in the other NAM families). As a consequence we considered the contrasts between each predicted individual and the mean of the family it belongs to. If the predicted NAM family i is composed of $\varvec{ N}_{\varvec{i}}$ individuals, we combined the $\varvec{ N}_{\varvec{i}}$ contrasts in a contrast matrix $\varvec{T}_{\varvec{i}} \varvec{ }$: each column of $\varvec{T}_{\varvec{i}}$ is a contrast between an individual of the NAM family i and the mean of this NAM family i. Dimensions of $\varvec{T}_{\varvec{i}}$ are: total number of individuals in the G-BLUP model (size of the calibration set + size of the predicted NAM family) x number of individuals in the predicted NAM family i.

We estimated the expected prediction accuracy in the NAM family *i* by: $\varvec{CDpop}_{\varvec{i}} = \frac{1}{{\varvec{N}_{\varvec{i}} }}\sum \left[ {\varvec{CD}\left( {\varvec{T}_{\varvec{i}} } \right) } \right]^{1/2}$, where $\varvec{T}_{\varvec{i}}$ is the contrast matrix of NAM family *i*, $\varvec{CD}\left( {\varvec{T}_{\varvec{i}} } \right)$ is the vector of the $\varvec{ N}_{\varvec{i}}$ CDs, corresponding to the $\varvec{ N}_{\varvec{i}}$ contrasts. We considered the average over the *N*_*i*_ individuals of the square root of the CDs to build the criterion $\varvec{CDpop}_{\varvec{i}}$, to make it consistent with prediction accuracy (defined as the square root of the reliability, or equivalently as the correlation between true and predicted genetic values).

### Using CD to estimate prediction reliability in structured populations

When breeders have collected phenotypes to run GS, they are interested in estimating the prediction reliability they can expect in different target populations. The CD is the expected reliability of predictions, and so is a good candidate criterion to help breeders evaluate their prediction accuracies.

We tested the efficiency of the CD (${\text{CDpop}}$) to predict GS accuracy in different scenarios:

- S1: one NAM family is predicted using another NAM family (scenario noted CwC in Lehermeier et al. 2014, for cross-with-cross predictions)
- S2: one NAM family is predicted using all other NAM families (scenario noted LOCO in Lehermerier et al. 2014, for leave-one-cross-out)
- S3: one NAM family is predicted by the panel of the same heterotic group. For S3 we also considered the case when the objective is to predict jointly all the NAM families as if it was an unstructured population (the CD criterion in this case was adapted and further referred to as CDallNAM).

For each situation we compared $${\text{CDpop}}$$ and the observed prediction accuracy estimated as the correlation between the predictions and the phenotypes divided by the square root of the heritability of the predicted family (estimated at the experimental design level, as presented in Lehermeier et al. 2014). The comparison was made on accuracies rather than reliabilities to keep track of possible negative correlations which would have generated positive reliabilities. In these three scenarios, we assumed that the phenotypes of the calibration set are available when CDpop is computed, we therefore used for BLUP predictions and CDpop computations a $$\lambda$$ value specific to each trait and each calibration set using the heritabilities ($$h^{2}$$) of the calibration set: $$\lambda = {\raise0.7ex\hbox{$1$} \!\mathord{\left/ {\vphantom {1 {h^{2} }}}\right.\kern-0pt} \!\lower0.7ex\hbox{${h^{2} }$}} - 1$$, as computed by Lehermeier et al. (2014) for the NAM families and Rincent et al. (2014a, b) for the panels.

### Using generalized CD to optimize calibration set in structured populations

As generalized CD can be computed before collecting phenotypes, it can be used to define optimal calibration set before field experimentation, provided genotypes are available. We tested if the generalized CD used with adapted contrasts (${\text{CDpop}}$) is efficient to define optimal calibration set to conduct GS in structured material such as NAM populations. For this, we used ${\text{CDpop}}$ to sample calibration sets of variable sizes (from 10 to 500 depending on the scenario) considering different optimization targets (OT):

- OT1: one NAM family is predicted using calibration sets of size 10, 50, 150, 300 or 500 sampled among the other NAM families
- OT2: one NAM family is predicted using calibration sets of size 10, 25, 50, 100 or 200 sampled from the panel of the corresponding heterotic group (CF-Dent for Dent NAM, and CF-Flint for the Flint NAM)
- OT3: all NAM families are predicted using calibration sets of size 50, 150, 300 or 500 sampled from all NAM families
- OT4: all NAM families are predicted using calibration sets of size 10, 25, 50, 100 or 200 sampled from the panel of the corresponding heterotic group (CF-Dent for Dent NAM, and CF-Flint for the Flint NAM)

In scenarios OT1 and OT2, the calibration set sampled to maximize CDpop was compared to calibration sets that were sampled randomly, or maximizing the average relatedness between calibration set and the predicted set (Crit_Kin). Crit_Kin was defined as the average of the relatedness coefficients between each individual in the calibration set and each predicted individual.

Additional sampling strategies were tested for scenario OT3: (1) stratified sampling without taking family sizes into account (Crit_Strat, i.e., a same number of individuals was sampled randomly in each family), (2) stratified sampling taking family sizes into account (Crit_Strat_size, the contribution of each family to the calibration set is weighted by its size), (3) the average of the CDpop of the ten NAM families (CDpop_mean), and (4) CDallNAM which is the average of the square root of the CD of the contrasts between each individual and the mean of all NAM individuals (the structure is not taken into account). For OT4 (calibration with a panel) we considered the same strategies of sampling as for OT3 except the Crit_Strat and Crit_Strat_size strategies, that do not make sense since the panels are not structured in clear subpopulations. This was done 20 times for each calibration set size. We then compared the observed prediction accuracies obtained with the different calibration sets. In OT3 and OT4, we computed the prediction accuracies within each NAM family and in the whole NAM population as if it was an unstructured population. In these four optimization situations (OT1, OT2, OT3 and OT4) we considered that phenotypes are not available when CDpop (or CDpop_mean) is computed and therefore set $\lambda$ to an arbitrary value of 1, corresponding to an intermediate heritability of 0.5. The maximal size of the calibration sets considered here (200–500 depending on the scenario) were constrained by the size of the dataset.

To sample calibration sets maximizing ${\text{CDpop}}$, CDallNAM or CDpop_mean, we used a simple exchange algorithm as in Rincent et al. (2012). At each step the random exchange of one individual between the calibration set and the set of individuals excluded from the calibration set was accepted if the criterion was improved, and was rejected otherwise. More complex algorithms did not give significantly better results and needed more iterations to converge. They were therefore not retained for further investigations. All scripts were written in R 2.14.0, the script to compute and optimize CDpop is available as supplementary information.

### Genetic properties of optimized calibration sets

To understand how the individuals selected to be part of the calibration set relate to the other individuals we used a network visualization of the genomic relationship matrix. We represented the individuals in a network, in which 2 individuals are linked when their relationship coefficient (*K*_ij_) is higher than a given threshold (0.2 for OT1 and OT3, 0.4 for OT2 and OT4), unlinked otherwise (Rozenfeld et al. 2008; Thomas et al. 2012). For this, the genomic relationship matrix was transformed in a matrix of Boolean indicating if the coefficients were above the threshold or not. The networks were drawn with a Fruchterman and Reingold’s force-directed placement algorithm (Fruchterman and Reingold 1991) with the package “network” in R 2.14.0.

## Results

### Using CD to estimate prediction reliability in multiparental populations

Highly variable observed accuracies were obtained in the S1 scenario (one NAM family predicted by each of the other NAM families) (Tables S1–S6). Average accuracies for the Dent and Flint NAM were of 0.40 and 0.31 for AD, 0.22 and 0.28 for DMY, 0.37 and 0.25 for PH, respectively. Depending on the trait and on the families observed, accuracies ranged from −0.55 to 0.82 in the Dent NAM and from −0.36 to 0.87 in the Flint NAM. The direction of prediction also influenced accuracy, for example, for AD the accuracy was of 0.52 when UH250 family predicted B73 family and of 0.24 when B73 family predicted UH250 family. These results are consistent with those presented in Lehermeier et al. (2014) (taking into account the fact that correlations were not divided by square root of heritability in their study).

In this S1 scenario, expected accuracy (CDpop) was also variable between families and traits (Tables 1, S7–S11). For example, in the Dent NAM families for AD, it ranged from 0.24 (UH304 family predicted by EC169 family) to 0.71 (D06 family predicted by D09 family), with an average value of 0.37.

**Table 1:** CDpop (expected reliability) computed with the ${\varvec{\uplambda}}$ estimated by REML for AD (Dent NAM families). The size of the families are indicated between brackets

		Predicted family										
		B73 (64)	D06 (99)	D09 (100)	EC169 (66)	F252 (96)	F618 (104)	Mo17 (53)	UH250 (94)	UH304 (81)	W117 (84)	Average
Calibration family	B73 (64)	.	0.30	0.27	0.42	0.27	0.30	0.29	0.31	0.25	0.28	0.30
	D06 (99)	0.41	.	0.67	0.48	0.40	0.42	0.38	0.66	0.39	0.39	0.47
	D09 (100)	0.40	0.71	.	0.42	0.42	0.41	0.39	0.62	0.45	0.39	0.47
	EC169 (66)	0.39	0.33	0.27	.	0.25	0.28	0.28	0.32	0.24	0.26	0.29
	F252 (96)	0.37	0.40	0.39	0.36	.	0.35	0.38	0.41	0.39	0.37	0.38
	F618 (104)	0.41	0.41	0.38	0.41	0.35	.	0.36	0.42	0.36	0.37	0.39
	Mo17 (53)	0.33	0.32	0.30	0.34	0.31	0.30	.	0.35	0.27	0.33	0.32
	UH250 (94)	0.42	0.66	0.58	0.45	0.41	0.41	0.41	.	0.37	0.38	0.46
	UH304 (81)	0.30	0.33	0.34	0.30	0.33	0.32	0.29	0.32	.	0.30	0.31
	W117 (84)	0.37	0.37	0.35	0.36	0.37	0.36	0.38	0.37	0.34	.	0.36
	Average	0.38	0.43	0.40	0.39	0.34	0.35	0.35	0.42	0.34	0.34	0.37

**Fig. 1:**
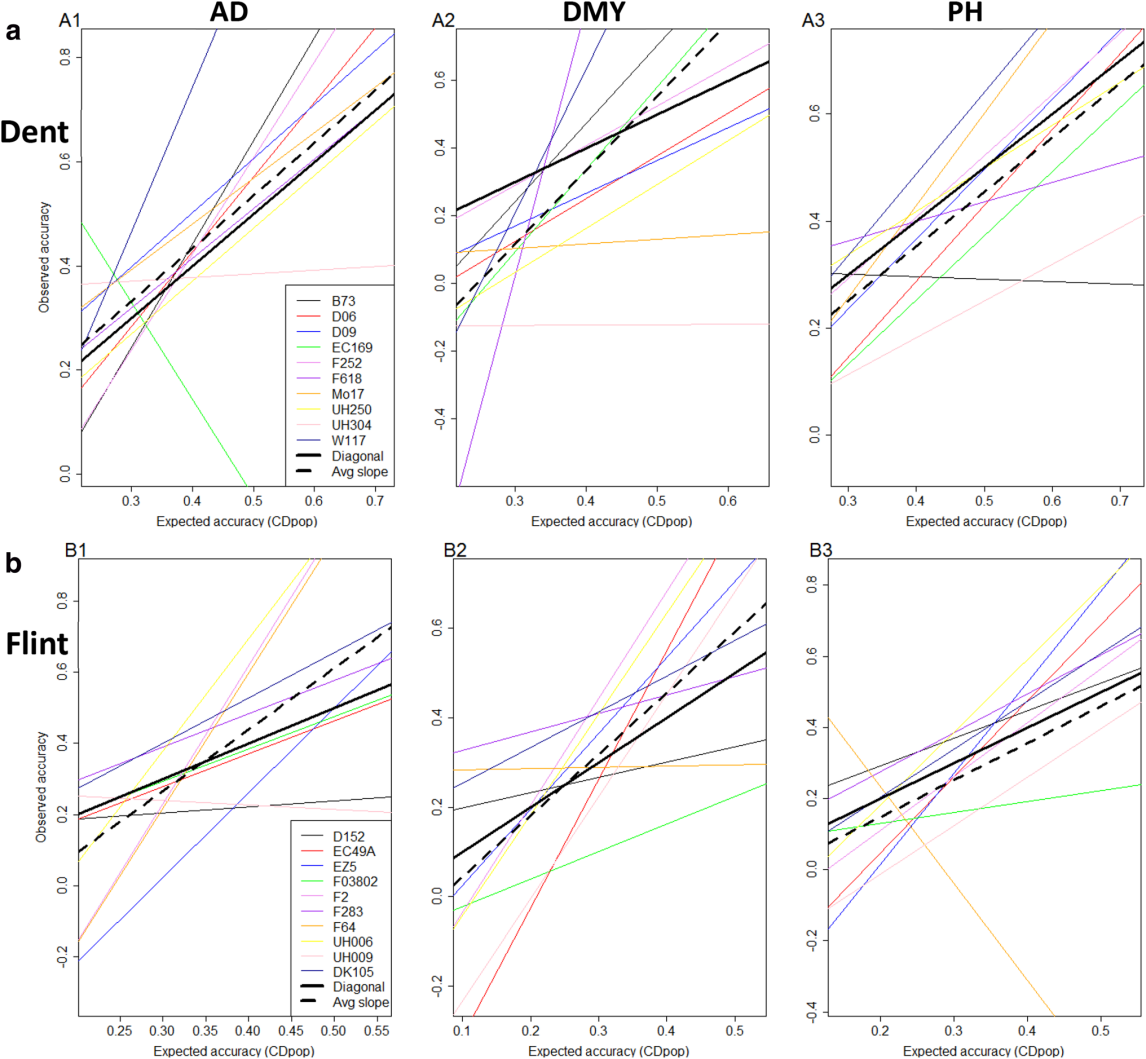
Expected (CDpop) and observed accuracies for scenario S1 (one NAM family is predicted by another NAM family) for the Dent (**a**) and the Flint (**b**) families, for AD (*A1* and *B1*), DMY (*A2* and *B2*) and PH (*A3* and *B3*). The regression of observed again expected accuracy is represented by a *line* for each predicted family. The *bold black line* is the *diagonal*, and the *dotted bold black line* is the average of the 10 regressions

The correlation between the expected accuracy (CDpop) and the observed accuracy was variable between traits and NAM populations (Fig. 1; Tables S12–S13). In both NAM populations, this correlation was higher for DMY (0.50 and 0.57 in the Dent and Flint NAM, respectively), than for AD (0.44 and 0.42) and PH (0.42 and 0.38). The averaged regression line of expected versus observed accuracies was close to the diagonal for both populations and all traits, except for DMY in the Dent NAM and AD in the Flint NAM (dotted line on Fig. 1). The correlation between observed and expected accuracy was variable between families. For example, for AD in the Flint NAM, correlation between CDpop and observed accuracy ranged from −0.03 (UH009) to 0.72 (F2). However, this correlation was positive for most families.

In the S2 scenario, in which nine of the ten families are used to predict the last one, observed accuracies (Table 2) were on average higher for AD (0.64 in the Dent population, and 0.61 in the Flint population), than for PH (0.60 and 0.55) and DMY (0.51 and 0.56). The accuracies were variable between crosses and traits. They ranged from −0.06 (DMY) to 0.85 (AD) in the Dent population (Table 2), and from 0.25 (PH) to 0.88 (AD) in the Flint population (Table 2). Expected accuracies (CDpop) overestimated the observed accuracies in 70 and 60% of the cases in the Dent and Flint families, respectively (Table 2). Correlations between expected and observed accuracies were variable between traits and NAM populations. It varied between 0.23 (AD) and 0.59 (PH) for the Dent NAM, and between 0.29 (DMY) and 0.61 (AD) for the Flint NAM.

**Table 2:** Observed and expected (CDpop) accuracy for scenario S2 for the Dent and Flint NAM families. In scenario S2 each NAM family is predicted by all other NAM families of the same NAM population. The size of the families are indicated between brackets

	Predicted family										Average	Correlation^a^
	Dent NAM											
	B73 (64)	D06 (99)	D09 (100)	EC169 (66)	F252 (96)	F618 (104)	Mo17 (53)	UH250 (94)	UH304 (81)	W117 (84)		
Accuracy AD	0.52	0.85	0.81	0.24	0.57	0.62	0.7	0.7	0.57	0.81	0.64	0.23
CDpop AD	0.7	0.84	0.79	0.72	0.62	0.62	0.62	0.8	0.63	0.61	0.7	
Accuracy DMY	0.62	0.64	0.63	0.67	0.64	0.59	0.31	0.55	-0.06	0.56	0.51	0.39
CDpop DMY	0.68	0.83	0.78	0.71	0.6	0.61	0.61	0.78	0.62	0.6	0.68	
Accuracy PH	0.49	0.81	0.72	0.43	0.64	0.59	0.54	0.81	0.34	0.64	0.6	0.59
CDpop PH	0.71	0.86	0.82	0.74	0.64	0.64	0.64	0.81	0.65	0.62	0.71	

	Flint NAM										Average	Correlation^a^
	D152 (72)	EC49A (29)	EZ5 (26)	F03802 (129)	F2 (54)	F283 (133)	F64 (64)	UH006 (94)	UH009 (98)	DK105 (95)		
Accuracy AD	0.4	0.48	0.39	0.58	0.7	0.74	0.61	0.88	0.61	0.72	0.61	0.61
CDpop AD	0.67	0.61	0.62	0.69	0.67	0.74	0.59	0.71	0.68	0.68	0.67	
Accuracy DMY	0.44	0.48	0.59	0.26	0.76	0.71	0.55	0.7	0.51	0.62	0.56	0.29
CDpop DMY	0.62	0.59	0.6	0.65	0.64	0.69	0.57	0.67	0.63	0.64	0.63	
Accuracy PH	0.6	0.55	0.65	0.31	0.53	0.76	0.25	0.77	0.43	0.67	0.55	0.5
CDpop PH	0.65	0.6	0.61	0.67	0.66	0.71	0.58	0.69	0.66	0.66	0.65	

^a^ Correlation between observed and expected (CDpop) accuracy for each trait

In the S3 scenario when each of the 10 NAM families is predicted by the panel of the same heterotic group, observed accuracies varied according to the trait and the NAM family considered (Tables S14–S15). For the two heterotic groups, observed accuracies were higher for AD. When considering the NAM families as a single population, prediction accuracies based on the corresponding panel were medium to high and varied between 0.43 and 0.72. However, prediction accuracies within family were much lower on average over the ten families (between 0.06 for DMY in the Dent population and 0.41 for AD and PH in the Flint population) and varied a lot from one family to the other (for example, from −0.23 to 0.34 for DMY in the Dent population). Within family accuracies were particularly low for the Dent heterotic group. Expected accuracy predicted by CD criteria were low (Tables S14–S15) and much lower than those reported in scenarios S1 and S2, when calibrations are based on NAM families. Expected accuracies usually overestimated observed accuracies when considering as test set either all NAM families (CDallNAM) or individual NAM families (CDpop). Correlations between observed and expected accuracies were small and varied between 0.12 for DMY in the Flint population and 0.45 for PH in the Dent population. They were close to zero or even negative when considering only within NAM family accuracies (the correlation varied between −0.32 for PH in the Flint population and 0.17 for PH in the Dent population). So CDpop appears inefficient to predict differences in within family accuracies when calibrations are based on panels, beyond the fact that there were all expected to be small.

### Using generalized CD to optimize calibration set in structured populations

**Fig. 2:**
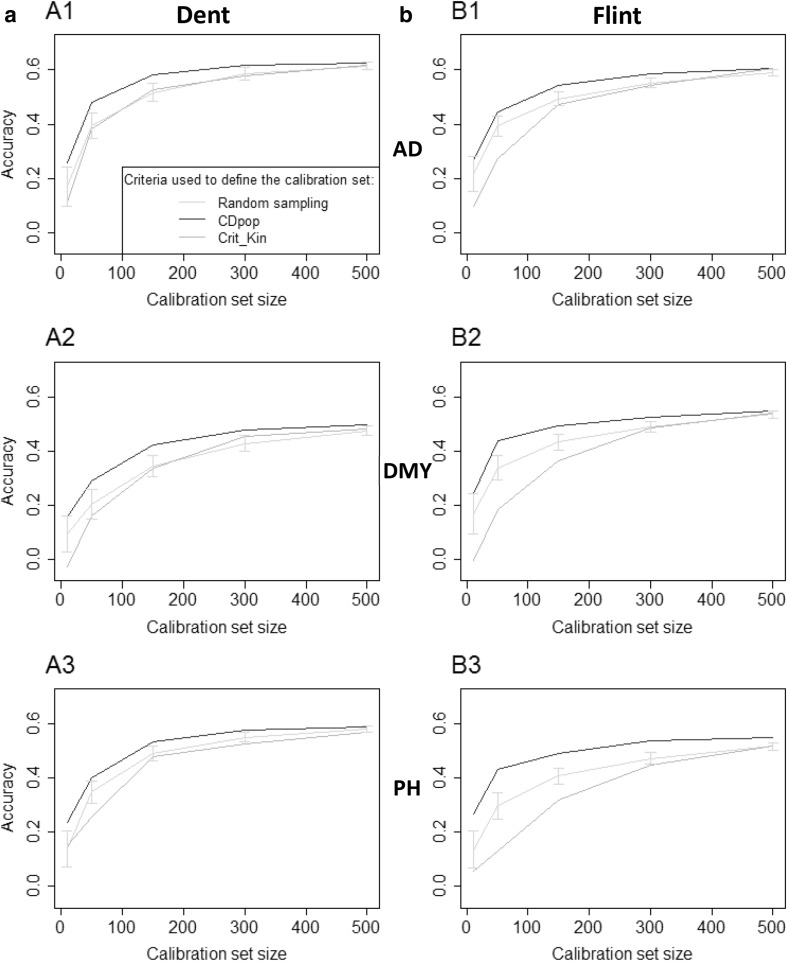
Observed accuracy (scenario OT1) obtained with the different calibration sets, for the different traits (*A1* and *B1*: AD, *A2* and *B2*: DMY, *A3* and *B3*: PH) in the two NAM populations (**a** Dent, **b** Flint). The accuracies were averaged over the 10 families. In OT1 the calibration set is sampled among nine families to predict one NAM family. The intervals indicated for the random samples correspond to an interval of two standard deviations (computed as the average of the standard deviations of the 10 families)

In the OT1 optimization, there was as expected a clear trend for the observed prediction accuracy to increase with the calibration set size for all traits in both populations (Fig. 2) except family UH304 for DMY (Fig. S1). For this family, accuracies for DMY were close to zero or even negative for all calibration set sizes and all sampling approaches. The particular behavior of this family was pointed out in Lehermeier et al. (2014, 2015). The accuracies obtained for the different families were highly variable, with families accurately predicted (for example, family D06 for AD in the Dent population), or poorly predicted even for calibration sets composed of 500 individuals (for example, EC169 for AD in the Dent population).

On average over the ten families in both Dent and Flint NAM populations, the calibration set optimized with CDpop always performed better than Crit_Kin and the random samples. Calibration set sampled with Crit_Kin was similar to or worse than random sampling. The ranking of the sampling approaches varied between predicted families, with CDpop doing as good as or better than random sampling for at least 7 of the 10 families in the Dent population, and 9 of the 10 families in the Flint population.

**Fig. 3:**
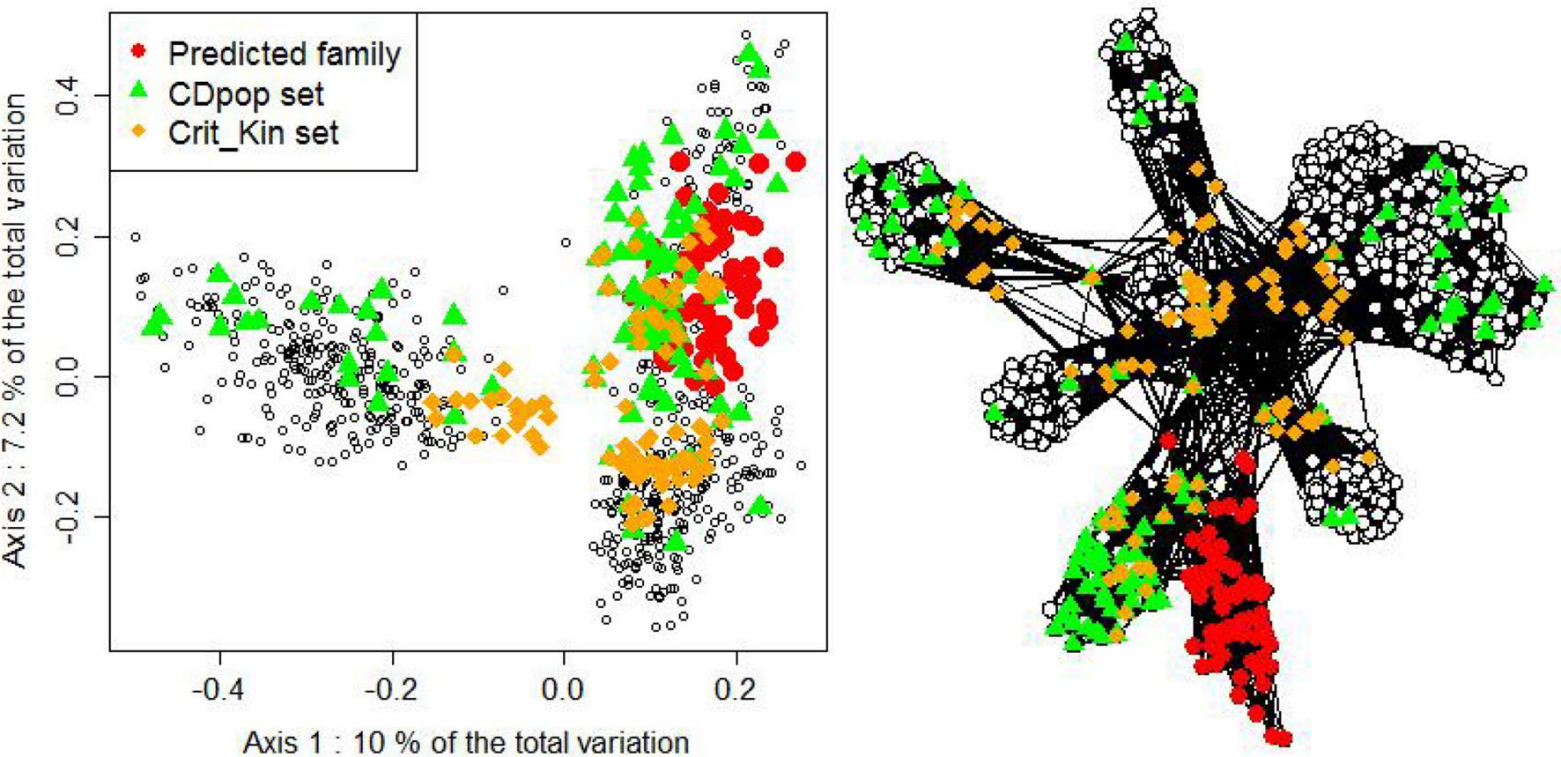
Principal coordinates analysis and network representation of the Dent NAM design. The calibration sets of size 100 obtained by maximizing CDpop or Crit_Kin are represented by* green triangles* and* orange squares*, respectively. The family to predict is represented by* red dots* (color figure online)

The PCoA and network visualizations of the NAM population (Dent population in Fig. 3) illustrate the different trends of the sampling algorithms for scenario OT1. Crit_Kin tended to sample individuals related to the predicted family and also related to each other. The individuals sampled by CDpop were also related with the predicted family, but relatedness was lower within the calibration set, allowing more distant individuals to be sampled (Fig. 3).

**Fig. 4:**
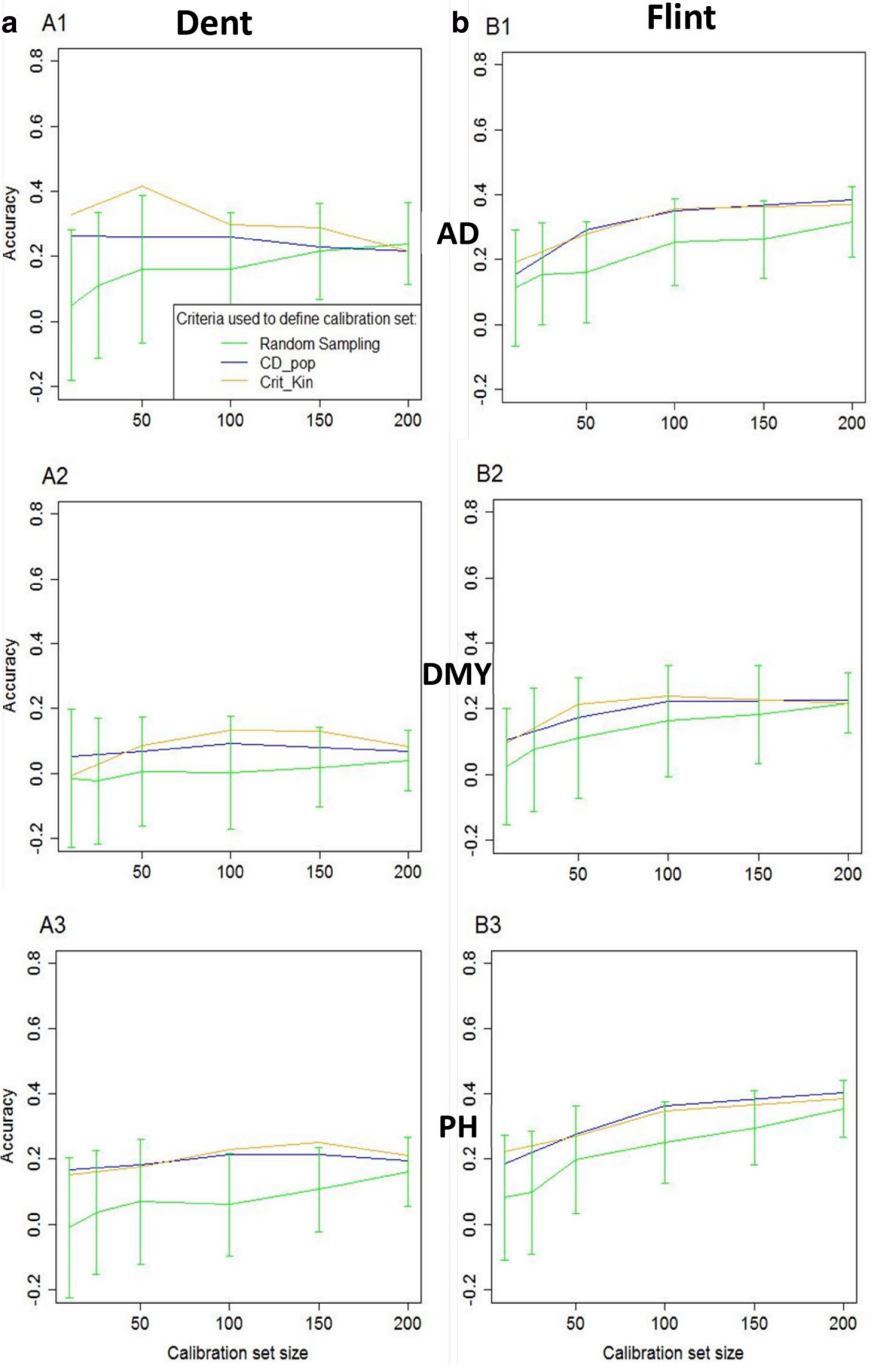
Observed accuracy (scenario OT2) obtained with the different calibration sets, for the different traits (*A1* and *B1*: AD, *A2* and *B2*: DMY, *A3* and *B3*: PH) in the two NAM populations (**a** Dent, **b** Flint). The accuracies were averaged over the 10 families. In OT2 the calibration set is sampled from the highly diverse panels to predict one NAM family. The intervals indicated for the random samples correspond to an interval of two standard deviations (computed as the average of the standard deviations of the 10 families)

**Fig. 5:**
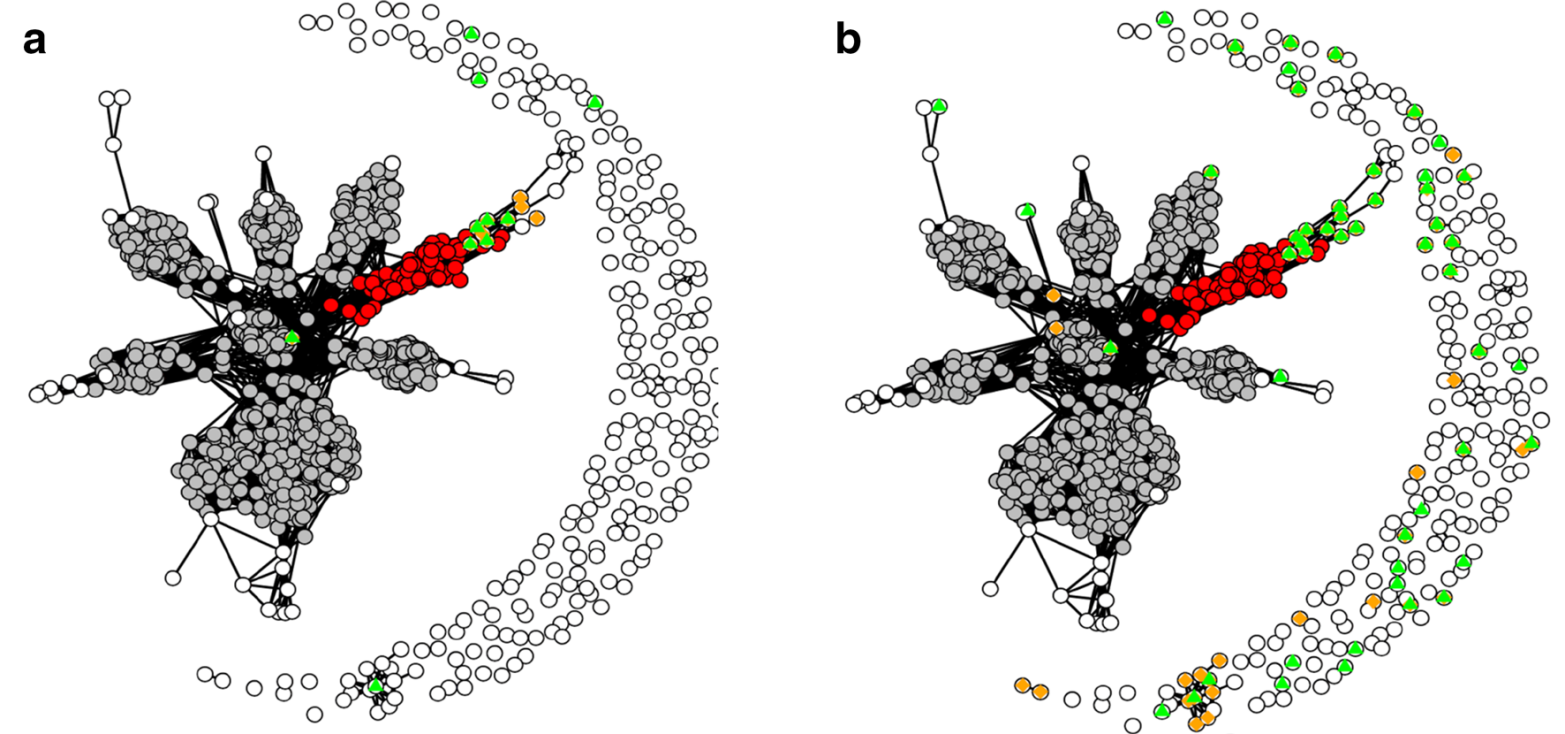
Sampling of calibration sets from the dent panel to predict one dent NAM family. The calibration sets of size 10 (**a**) and 50 (**b**) obtained by maximizing CDpop or Crit_Kin are represented by *green triangles* and *orange squares*, respectively. The family to predict is represented by *red dots*. The other NAM families and the *lines* included in the panel are represented by gray and *white dots*, respectively. The *network connects lines* that have a kinship >0.4. Only few lines from the panel are connected to each NAM family (color figure online)

In the OT2 scenario in which individuals from the panel are sampled to best predict a given NAM family, the accuracies of prediction increased with the calibration set size especially when calibration sets are sampled at random (Fig. 4) but prediction accuracies remained low whatever the calibration set size considered. On average over the ten NAM families, optimization of the calibration set either using CDpop or Crit_Kin improved the accuracy of prediction compared to using random samples of the panel as calibration set in almost all cases. However, contrary to what was observed in the optimizations OT1, CDpop did not outperform Crit_Kin,. This is consistent with both methods sampling almost the same individuals in the panel (differences between selected calibration samples were only noticeable for very small calibration set sizes, Fig. 5). Prediction accuracies with calibration sets optimized either with Crit_Kin or CDpop were almost stable for calibration set sizes over 50 individuals, especially in the Dent group.

**Fig. 6:**
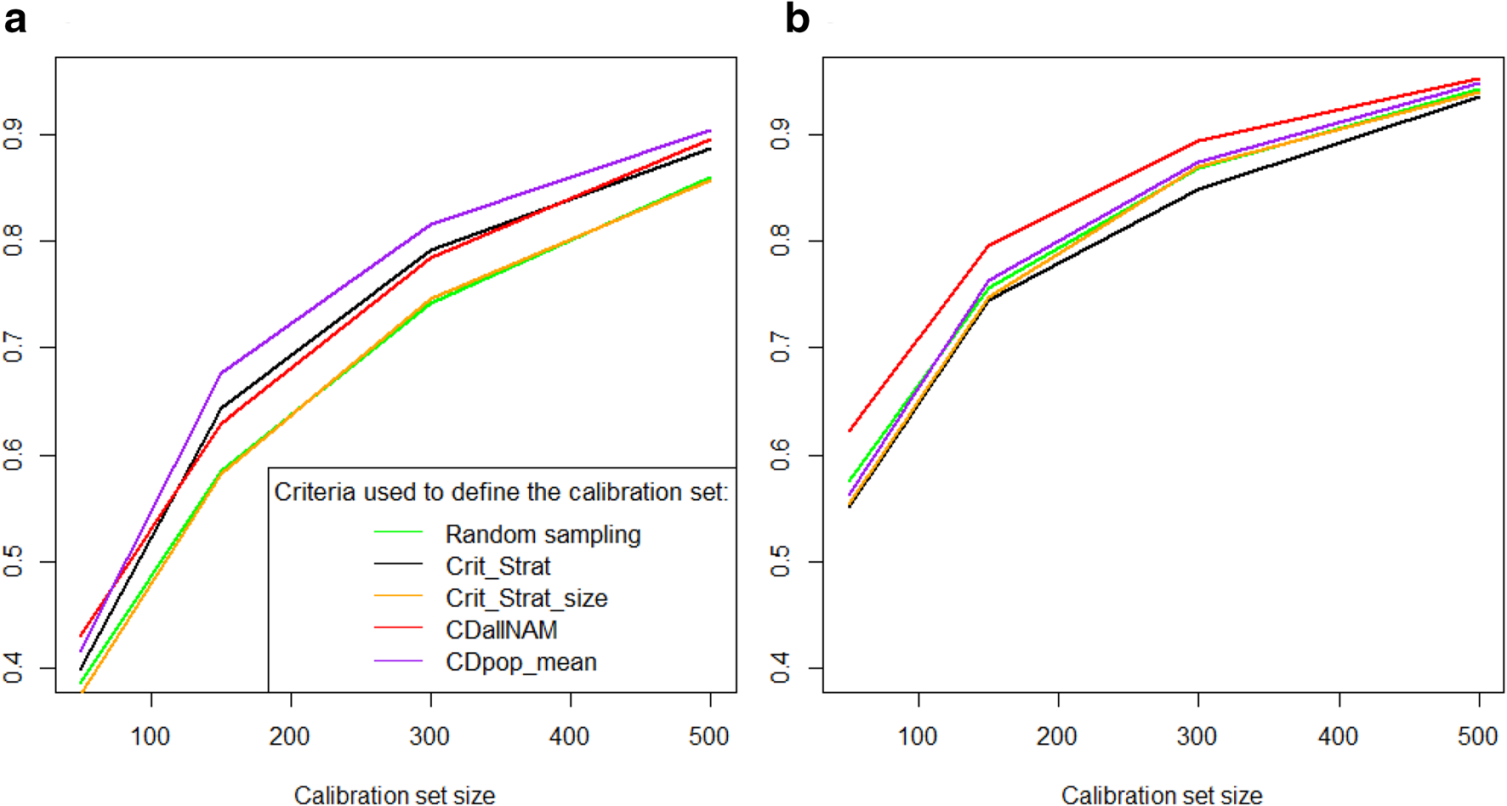
Observed accuracies obtained in scenario OT3 for PH in the Flint population for calibration set sizes of 50, 150, 300 and 500 individuals. In OT3 the calibration set is sampled among all families to predict all families simultaneously. Accuracies are then computed for intra-family predictions (**a**, average over the 10 families) and for global predictions as if it was an unstructured population (**b**)

In the OT3 optimizations, in which NAM individuals are sampled in all NAM families to predict all families, all sampling strategies resulted in similar trends. The average over the 10 families of observed accuracy increased with calibration set size (Figs. 6, S2). Within family prediction accuracies were higher in OT3 than in OT2, where individuals in calibration were not sampled in the predicted family. Observed accuracies were lower for within family predictions than for the global prediction of the whole population. Random sampling resulted most of the time in the worst predictions. The best calibration set was always sampled using a criterion derived from CD (CDallNAM or CDpop_mean). As expected, CDpop_mean tended to produce better predictions within families, whereas CDallNAM was more efficient for global predictions (simultaneously in all families as if it was a single unstructured population). Crit_Strat also increased accuracy in comparison to random sampling, but Crit_Strat_size performed similarly to random sampling.

**Fig. 7:**
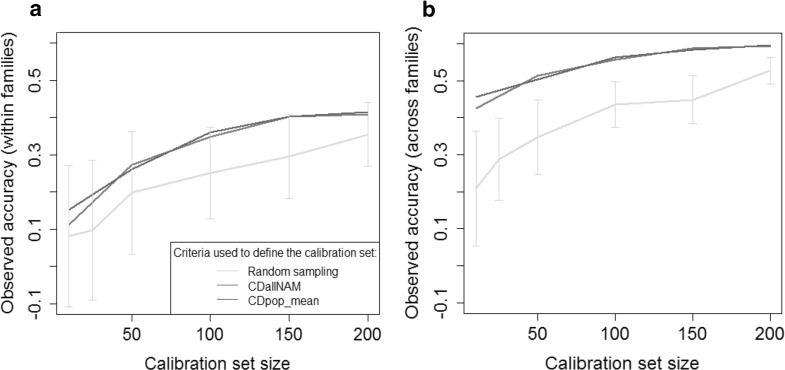
Observed accuracies obtained in scenario OT4 for PH in the Flint population. In OT4 the calibration set is sampled in the highly diverse panel to predict all NAM families simultaneously. Accuracies are then computed for intra-family predictions (**a**) and for global predictions as if it was an unstructured population (**b**). The intervals indicated for the random samples correspond to an interval of two standard deviations (computed as the average of the standard deviations of the 10 families for graph A)

In the OT4 optimizations (Figs. 7, S3), when the objective is to predict jointly all NAM families using panels, we observed that all optimization methods outperformed the “random” selection strategy. Prediction accuracies were higher when the objective was to predict values of the NAM population as a whole rather than to predict within family performances. Differences between optimization strategies were small. When the objective was to predict jointly all NAM families, optimization based on CDallNAM gave on average slightly better predictions than optimizations based on CDpop_mean. When the objective was to predict values within each of the NAM families, CDpop_mean gave on average slightly better predictions.

## Discussion

### CD criteria could partly explain prediction reliability in multiparental populations

In traditional plant breeding programs, candidates are often evaluated in the same trial network and the accuracy of the estimation of their genetic merit based on performances is generally identical. On the contrary, in animal breeding, the accuracy of the breeding value predictions can vary a lot from one individual to the other, taking values close to 0 to values close to 1 (depending on the number of relatives that have been phenotyped). In this case, selection is based on the predicted breeding value of candidates but also on the accuracy of these predictions. Considering prediction accuracy at the level of individuals is especially important in genomic selection of both animals and plants, since predictions can be made for any genotyped individual, and the reliability of the predictions can vary a lot depending on the relatedness between the candidates and the individuals used for calibration. The criterion used to estimate the accuracy depends on the selection objective. For instance in plants, selected populations are structured in biparental populations. The main objective of the breeders is often to identify the best individuals within each biparental population. We therefore evaluated different criteria to estimate prediction accuracy in such types of structured populations. In particular we adapted the CDmean criterion (Rincent et al. 2012) to these situations by considering the contrast between each individual of the predicted family and the average of its family (CDpop).

In the S1 and S2 prediction scenarios (S1: one NAM family predicted by another NAM family, or S2: by all the other NAM families), the expected accuracy of the predictions estimated with CDpop was variable between traits and families (Tables 1, S7–S11). In the S1 scenario, CDpop tended to be (as expected, and in accordance with the observed accuracies) higher for larger calibration families (D06, D09, F618 for the Dent, F03802 and F283 for the Flint), and when calibration and predicted family are highly related (for example, D06 and D09 for the Dent, UH006 and UH009 for the Flint). The size of the calibration set and its relatedness to the predicted set are known to be key parameters influencing observed prediction accuracy (Habier et al. 2010; Albrecht et al. 2011; Pszczola et al. 2012). In the S1 scenario, CDpop was able to partly predict the variation of observed prediction accuracies, with correlations between expected (CDpop) and observed accuracy averaged over the ten families ranging from 0.42 to 0.57 (Tables S12–S13). These levels of correlations are encouraging when considering the average over the ten families. But correlations between expected and observed accuracies were highly variable from one target family to another (Fig. 1; Tables S12–S13) and sometimes negative. This means that the efficiency of CDpop to estimate the levels of prediction accuracies highly depends on the families considered. In the S2 scenario, CDpop could also partly explain the variability of the prediction accuracies (average correlations between 0.23 and 0.61, Table 2). But again these correlations were quite variable between traits and the two NAM populations, and CDpop often overestimated the observed accuracies. This means that CDpop is helpful to identify which families can be efficiently or poorly predicted, but is not very precise to evaluate the absolute level of accuracies.

In the case where panels were used to predict NAM families (S3 scenario), we observed small accuracies of prediction for all individual NAM families and a higher one when considering the NAM families as a single population (Tables S14–S15). The higher prediction accuracies obtained when considering globally the NAM populations rather than within family predictions illustrate (i) the good ability of GS models to predict the average performance of a biparental family when the parental inbred lines and/or close relatives are included in the calibration set (as it is the case here) and (ii) that it is more difficult to predict the value of individuals within families (i.e., the variation due to mendelian sampling). The CD criterion was efficient to predict that within family accuracies (CDpop) would be lower than global accuracy (CDallNAM). In the S3 scenario, the observed accuracies were variable from one NAM family to the other as in S2 but this variation was not correlated with the variation of the CDpop criterion. This means that for situations with low prediction accuracies (between 0.06 and 0.41) it was not possible to predict the differences of accuracies between families.

One thing that probably limits the adequacy between expected and observed accuracy in scenarios S1, S2 and S3, is that the $\lambda$ values were estimated with the phenotypes of the calibration set (which are the only phenotypes available to breeders). If the size of the calibration set is reduced, $\lambda$ will be poorly estimated and as a result CDpop could strongly over- or underestimate the observed accuracy. The other important point is that the true $\lambda$ values could potentially be different in the calibration set and in the predicted family (especially in the S3 scenario when calibration is done in a panel and validation is done in a NAM family). We also have to consider that the observed accuracies were computed using estimates of heritability which can also be poorly estimated, particularly in the families of small size. In that case observed accuracy can deviate from the true (but inaccessible) accuracy, leading to artificial inadequacy between expected and observed accuracies. One other limit to the use of CD criteria in structured populations is that different QTL may segregate in the different populations. In these situations the relatedness matrix may poorly reflect the genetic covariance between individuals of different families, resulting in poor estimates of expected accuracy. This may explain the low correlation between observed and expected accuracy obtained when predicting UH304 and Mo17 (for DMY) families in scenario S1 (Table S12). Mo17 gives a poor performing hybrid when crossed to the Flint tester used to evaluate the Dent NAM population. This suggests that compared to other Dent parental inbred lines it may share more unfavorable alleles with the Flint. Recently Lehermeier et al. (2015) tested different GS models taking into account family structure in the Dent NAM population, including models assuming marker effects specific of each family. They did not find any advantage for most of the families except the UH304 families, which corroborates the hypothesis of specific QTL effects. Note also that this population results from the cross between the two closest parents (as illustrated by its central position in Fig. S4).

### Calibration sets can be optimized to make predictions in structured populations

The CDmean criterion (Rincent et al. 2012) has proven efficient in sampling optimal calibration sets in highly diverse populations (Cros et al. 2015; Tayeh et al. 2015). We tested here the efficiency of CDpop for optimizing the composition of the calibration set in the context of highly structured populations. This criterion takes into account the structuration in subpopulations by considering adapted contrasts. In scenarios OT1 and OT2, when looking at prediction accuracies averaged over the ten NAM families, CDpop always resulted (for all traits and all calibration set sizes) in higher accuracies than random sampling (Figs. 2, 4). For example, for PH in the Flint NAM with calibration sets of 50 individuals in scenario OT1, the average accuracy obtained with calibration sets sampled with CDpop was 0.43, and only 0.27 for random samples (Fig. 2). It was also superior to the sampling based on kinship (Crit_Kin) for scenario OT1 and similar for scenario OT2. The different sampling strategies performed similarly for the largest calibration set size (500) in scenario OT1, probably because in this situation the overlap between the calibration sets was very important (as revealed by the small variability of accuracies obtained with the random samples). The accuracies obtained for individual families were quite variable, but CDpop outperformed random sampling on average over the families in all the situations considered. In scenario OT1, accuracies obtained with calibration sets maximizing relatedness with the predicted family performed poorly, and most of the time did worse than random sampling. This may be because the individuals composing these calibration sets could have important levels of relatedness between them (Fig. 3). This trend was not observed in optimizations OT2 (Fig. 4), when calibration sets were selected in a panel, probably because the panels are composed of individuals chosen to be as unrelated to each other as possible. In this situation, CDpop and Crit_Kin sampled almost the same individuals (Fig. 5). It is interesting to note that, in this situation, the observed accuracies of prediction were only poorly improved by adding individuals to the calibration set (in some cases they even decreased). This suggests that once the NAM parental inbred lines and the few inbred lines related to them are included in the calibration set, there is little interest in adding to the calibration set less related individuals (see Fig. 5 for the position of selected individuals in the relatedness network).

In the OT3 and OT4 scenarios, we predicted the ten NAM families simultaneously by sampling individuals from the NAM families (OT3) or from the panel of the corresponding heterotic group (OT4), and we considered two accuracies: the global accuracy (as if the NAM population was homogeneous without any structure in subpopulations), and the average of the ten within family accuracies. We used two different criteria to maximize these two accuracies. CDallNAM was used with the objective of maximizing the global accuracy (as it was developed in the context of unstructured panels), and another criterion (CDpop_mean, which is the average of CDpop over the ten families) was used with the objective of maximizing the average of the ten within family accuracies. These two criteria derived from the CD theory consistently lead to the most efficient calibration sets for all traits in both NAM populations (Figs. 6, 7, S2, S3). CDallNAM was often the best sampling strategy to reach high global accuracy, whereas CDpop_mean was more efficient to reach high within family accuracies. This confirms the fact that it is essential to consider appropriate contrasts when computing criteria related to CD. This proves that different criteria derived from the CD theory can be defined to reach different objectives. But it also means that depending on the objective, the optimal calibration set can potentially be different.

### How to increase the efficiency of CD criterion to estimate prediction accuracies and optimize calibration sets?

One key message of this study is that depending on the objective, one has to consider different contrasts to predict accuracies with criteria derived from CD. Once an appropriate criterion has been chosen, it can be used efficiently to optimize calibration set(s) adapted to the targeted populations.

The size of the calibration sets considered in our optimization process (<500) are moderate in comparison to the datasets that are used by breeders. Considering different selection cycles simultaneously can indeed result in accumulating information on thousands of individuals. It has been shown that building across cycle calibration sets could significantly increase prediction accuracy when the cycles are connected by common ancestors (Auinger et al. 2016). In this case both genotypic and phenotypic information is available to build the calibration set, and sampling criterion taking jointly both information into account (Rabier et al. 2016) would be helpful. For the calibration set sizes considered in the present study (constrained to a maximum of 500 because of the size of the dataset) we could sample the CD calibration sets within few seconds to few minutes, so computational time was not an issue. For high number of individuals (thousands to tens of thousands) the sampling of the CD calibration set may be too long. In that case faster sampling algorithms and other optimization criteria such as those proposed by Akdemir et al. (2015) or Bustos-Korts et al. (2016) might help reduce computational time. To our knowledge, however, these approaches are not adapted to optimize the accuracy of prediction of specific contrasts as done in this study. It would be therefore interesting to extend these approaches to the prediction of contrasts. Another possibility to reduce computational time would be to initialize the exchange algorithm with a relevant calibration set, for example, by maximizing relatedness between the calibration set and the test set.

Our results also revealed that criteria based on CD of appropriate contrasts were able to partly predict the accuracies obtained in different scenarios. However, we still need to increase the consistency between expected and observed accuracy to help breeders select candidates with known accuracies, especially when the calibration set and the predicted set are genetically distant or have different levels of structuration.

The criteria derived from CD all rely on the G-BLUP theory, and thus share its advantages and drawbacks. One consequence is that they are adapted to highly polygenic traits but would probably perform poorly for traits affected by few majors QTLs. This may explain why the correlation between expected and observed accuracy was low for AD in the Dent NAM population, because this trait is influenced by major QTLs (Giraud et al. 2014). In these situations other strategies have to be developed, for example, by considering major genes as fixed effect in the prediction model (Bernardo 2014) or by adapting the kinship estimation using the available knowledge on the genetic architecture (Rabier et al. 2016). As shown in other studies, the quality of the kinship estimate is indeed a very important element in genomic selection (and in association mapping) to make reliable predictions, and here to estimate CD criteria. Recently, Wientjes et al. (2015) proposed to modify the kinship estimates to account for differences in allelic frequencies between the calibration set and the family to predict and to explicitly take into account population structure when evaluating the expected efficiency of genomic predictions. This certainly deserves further investigation.

Another limit of the CD is that it always increases when individuals are added to the calibration set. This seems logical because adding new phenotypes also means adding additional information, but it is contradictory to our results and many observed situations in which prediction accuracies remained stable or even decreased when genetically distant individuals were added to the calibration set (Riedelsheimer et al. 2013; Lorenz and Smith 2015). This contradiction between theory and practice may again be explained by how kinship is estimated. Other kinship estimators using shrinkage (Müller et al. 2015) or kernels (Heslot and Jannink 2015) may be useful in this context. Lehermeier et al. (2015) adapted prediction models to take into account genetic heterogeneity in structured populations and found a benefit of using them in some highly structured populations. Estimating CD of suitable contrasts with these models and evaluating their ability to predict observed accuracies deserve further research.

#### **Author contribution statement**

RR and LM conducted statistical analyses; RR wrote the manuscript; LM and AC revised the manuscript critically.

## Electronic supplementary material

Below is the link to the electronic supplementary material.

Supplementary material 1 (DOCX 1731 kb)
