# Supplementary material for: Predicting genomic selection efficiency to optimize calibration set and to assess prediction accuracy in highly structured populations
Source: Theor Appl Genet. 2017 Aug 9;130(11):2231–47. doi: 10.1007/s00122-017-2956-7 (PMC5641287; doi:10.1007/s00122-017-2956-7)
Supplement: Supplementary file 1 — Supplementary material 1 (DOCX 1731 kb) [file 122_2017_2956_MOESM1_ESM.docx]

**Supplementary information**

Table S1: Observed prediction accuracy (AD, Dent NAM families). Family of column k predicted with family of line j

|  |  | Predicted family | | | | | | | | | | |
| --- | --- | --- | --- | --- | --- | --- | --- | --- | --- | --- | --- | --- |
|  |  | B73 | D06 | D09 | EC169 | F252 | F618 | Mo17 | UH250 | UH304 | W117 | **Average** |
| Calibration  family | B73 | . | 0.32 | 0.35 | 0.16 | 0.19 | 0.46 | 0.46 | 0.24 | 0.28 | 0.45 | 0.32 |
|  | D06 | 0.33 | . | 0.70 | 0.01 | 0.30 | 0.40 | 0.27 | 0.60 | 0.11 | 0.65 | 0.37 |
|  | D09 | 0.51 | 0.81 | . | 0.08 | 0.50 | 0.46 | 0.38 | 0.61 | 0.47 | 0.79 | 0.51 |
|  | EC169 | 0.32 | 0.07 | 0.19 | . | 0.12 | 0.10 | 0.47 | 0.07 | 0.47 | 0.24 | 0.23 |
|  | F252 | 0.35 | 0.47 | 0.57 | 0.16 | . | 0.10 | 0.34 | 0.45 | 0.48 | 0.62 | 0.39 |
|  | F618 | 0.54 | 0.54 | 0.45 | 0.07 | 0.19 | . | 0.67 | 0.31 | 0.39 | 0.61 | 0.42 |
|  | Mo17 | 0.42 | 0.33 | 0.33 | 0.28 | 0.25 | 0.62 | . | 0.52 | 0.30 | 0.58 | 0.40 |
|  | UH250 | 0.52 | 0.82 | 0.73 | 0.05 | 0.49 | 0.43 | 0.61 | . | 0.29 | 0.70 | 0.52 |
|  | UH304 | 0.17 | 0.22 | 0.43 | 0.34 | 0.42 | 0.19 | 0.20 | 0.31 | . | 0.57 | 0.32 |
|  | W117 | 0.41 | 0.60 | 0.73 | 0.25 | 0.45 | 0.55 | 0.54 | 0.41 | 0.57 | . | 0.50 |
|  | **Average** | 0.40 | 0.47 | 0.50 | 0.16 | 0.32 | 0.37 | 0.44 | 0.39 | 0.37 | 0.58 | 0.40 |

Table S2: Observed prediction accuracy (DMY, Dent NAM families). Family of column k predicted with family of line j

|  |  | Predicted family | | | | | | | | | | |
| --- | --- | --- | --- | --- | --- | --- | --- | --- | --- | --- | --- | --- |
|  |  | B73 | D06 | D09 | EC169 | F252 | F618 | Mo17 | UH250 | UH304 | W117 | **Average** |
| Calibration  family | B73 | . | 0.28 | 0.29 | 0.55 | 0.38 | 0.48 | 0.25 | 0.04 | -0.20 | 0.43 | 0.28 |
|  | D06 | 0.44 | . | 0.43 | 0.29 | 0.24 | 0.41 | -0.19 | 0.44 | -0.11 | 0.44 | 0.26 |
|  | D09 | 0.57 | 0.51 | . | 0.04 | 0.29 | 0.70 | 0.06 | 0.35 | -0.15 | 0.36 | 0.30 |
|  | EC169 | 0.44 | 0.14 | 0.03 | . | 0.16 | 0.19 | 0.09 | 0.31 | -0.02 | 0.13 | 0.17 |
|  | F252 | 0.51 | 0.31 | 0.30 | 0.46 | . | 0.54 | 0.41 | 0.32 | -0.17 | 0.58 | 0.36 |
|  | F618 | 0.50 | 0.36 | 0.41 | 0.25 | 0.33 | . | 0.18 | 0.09 | -0.38 | 0.45 | 0.24 |
|  | Mo17 | 0.22 | -0.15 | 0.05 | 0.05 | 0.37 | 0.24 | . | -0.29 | -0.01 | 0.42 | 0.10 |
|  | UH250 | 0.23 | 0.51 | 0.42 | 0.46 | 0.45 | 0.33 | -0.34 | . | 0.11 | 0.14 | 0.26 |
|  | UH304 | -0.31 | -0.15 | -0.02 | -0.12 | 0.04 | -0.55 | 0.08 | 0.14 | . | -0.19 | -0.12 |
|  | W117 | 0.56 | 0.47 | 0.23 | 0.23 | 0.53 | 0.45 | 0.41 | 0.05 | -0.18 | . | 0.31 |
|  | Average | 0.35 | 0.25 | 0.24 | 0.25 | 0.31 | 0.31 | 0.11 | 0.16 | -0.12 | 0.31 | 0.22 |

Table S3: Observed prediction accuracy (PH, Dent NAM families). Family of column k predicted with family of line j

|  |  | Predicted family | | | | | | | | | | |
| --- | --- | --- | --- | --- | --- | --- | --- | --- | --- | --- | --- | --- |
|  |  | B73 | D06 | D09 | EC169 | F252 | F618 | Mo17 | UH250 | UH304 | W117 | **Average** |
| Calibration  family | B73 | . | 0.17 | 0.10 | 0.14 | 0.41 | 0.33 | 0.58 | 0.16 | 0.05 | 0.58 | 0.28 |
|  | D06 | 0.22 | . | 0.69 | 0.39 | 0.43 | 0.32 | 0.33 | 0.64 | 0.24 | 0.62 | 0.43 |
|  | D09 | 0.24 | 0.69 | . | 0.28 | 0.51 | 0.28 | 0.20 | 0.57 | 0.10 | 0.50 | 0.37 |
|  | EC169 | 0.24 | 0.37 | 0.47 | . | 0.28 | 0.50 | 0.33 | 0.58 | -0.01 | 0.35 | 0.35 |
|  | F252 | 0.48 | 0.49 | 0.49 | 0.28 | . | 0.48 | 0.52 | 0.43 | 0.20 | 0.64 | 0.45 |
|  | F618 | 0.31 | 0.34 | 0.49 | 0.39 | 0.50 | . | 0.51 | 0.63 | 0.26 | 0.33 | 0.42 |
|  | Mo17 | 0.46 | 0.12 | 0.20 | 0.22 | 0.32 | 0.49 | . | 0.52 | 0.21 | 0.31 | 0.32 |
|  | UH250 | 0.30 | 0.73 | 0.73 | 0.62 | 0.36 | 0.65 | 0.64 | . | 0.27 | 0.44 | 0.53 |
|  | UH304 | -0.06 | 0.11 | 0.23 | 0.03 | 0.21 | 0.31 | 0.32 | 0.36 | . | 0.33 | 0.20 |
|  | W117 | 0.48 | 0.47 | 0.41 | 0.20 | 0.52 | 0.19 | 0.29 | 0.31 | 0.19 | . | 0.34 |
|  | Average | 0.30 | 0.39 | 0.42 | 0.28 | 0.39 | 0.40 | 0.41 | 0.47 | 0.17 | 0.45 | 0.37 |

Table S4 : Observed prediction accuracy (AD, Flint NAM families). Family of column k predicted with family of line j

|  |  | Predicted family | | | | | | | | | | |
| --- | --- | --- | --- | --- | --- | --- | --- | --- | --- | --- | --- | --- |
|  |  | D152 | EC49A | EZ5 | F03802 | F2 | F283 | F64 | UH006 | UH009 | DK105 | Average |
| Calibration  family | D152 | . | 0.31 | -0.09 | -0.08 | 0.16 | 0.19 | -0.17 | 0.46 | 0.68 | 0.25 | 0.19 |
|  | EC49A | 0.24 | . | -0.14 | 0.21 | 0.43 | 0.51 | 0.25 | 0.54 | 0.35 | 0.43 | 0.31 |
|  | EZ5 | -0.02 | 0.06 | . | 0.27 | -0.10 | 0.06 | 0.69 | -0.11 | -0.11 | 0.16 | 0.10 |
|  | F03802 | 0.25 | 0.21 | 0.38 | . | 0.70 | 0.44 | 0.45 | 0.57 | 0.18 | 0.44 | 0.40 |
|  | F2 | 0.11 | 0.35 | -0.08 | 0.66 | . | 0.63 | 0.31 | 0.48 | 0.06 | 0.52 | 0.34 |
|  | F283 | 0.23 | 0.64 | -0.06 | 0.47 | 0.63 | . | 0.33 | 0.87 | 0.47 | 0.70 | 0.48 |
|  | F64 | 0.08 | 0.17 | 0.59 | 0.45 | 0.41 | 0.37 | . | 0.25 | -0.20 | 0.49 | 0.29 |
|  | UH006 | 0.32 | 0.50 | -0.13 | 0.50 | 0.58 | 0.80 | 0.21 | . | 0.55 | 0.74 | 0.45 |
|  | UH009 | 0.46 | 0.01 | 0.07 | -0.05 | 0.06 | 0.28 | -0.32 | 0.45 | . | 0.15 | 0.12 |
|  | DK105 | 0.21 | 0.43 | 0.13 | 0.45 | 0.57 | 0.71 | 0.45 | 0.70 | 0.16 | . | 0.42 |
|  | Average | 0.21 | 0.30 | 0.07 | 0.32 | 0.38 | 0.44 | 0.25 | 0.47 | 0.24 | 0.43 | 0.31 |

Table S5 : Observed prediction accuracy (DMY, Flint NAM families). Family of column k predicted with family of line j

|  |  | Predicted family | | | | | | | | | | |
| --- | --- | --- | --- | --- | --- | --- | --- | --- | --- | --- | --- | --- |
|  |  | D152 | EC49A | EZ5 | F03802 | F2 | F283 | F64 | UH006 | UH009 | DK105 | Average |
| Calibration  family | D152 | . | 0.36 | 0.25 | 0.10 | 0.51 | 0.28 | 0.26 | 0.29 | 0.39 | 0.31 | 0.31 |
|  | EC49A | 0.13 | . | 0.40 | -0.03 | -0.23 | 0.36 | 0.38 | 0.01 | -0.21 | 0.30 | 0.12 |
|  | EZ5 | 0.12 | 0.31 | . | -0.03 | 0.35 | 0.43 | 0.46 | 0.14 | -0.22 | 0.44 | 0.22 |
|  | F03802 | 0.27 | -0.03 | 0.23 | . | 0.40 | 0.17 | 0.20 | 0.18 | 0.04 | 0.26 | 0.19 |
|  | F2 | 0.40 | -0.05 | 0.34 | 0.18 | . | 0.39 | 0.30 | 0.42 | 0.24 | 0.45 | 0.30 |
|  | F283 | 0.23 | 0.51 | 0.50 | 0.18 | 0.53 | . | 0.35 | 0.69 | 0.40 | 0.64 | 0.45 |
|  | F64 | 0.25 | 0.38 | 0.62 | 0.07 | 0.37 | 0.33 | . | 0.22 | 0.13 | 0.42 | 0.31 |
|  | UH006 | 0.22 | 0.19 | 0.12 | 0.10 | 0.65 | 0.72 | 0.25 | . | 0.51 | 0.56 | 0.37 |
|  | UH009 | 0.40 | -0.19 | -0.20 | -0.02 | 0.32 | 0.28 | 0.05 | 0.44 | . | 0.08 | 0.13 |
|  | DK105 | 0.29 | 0.38 | 0.55 | 0.23 | 0.53 | 0.68 | 0.35 | 0.58 | 0.06 | . | 0.40 |
|  | Average | 0.26 | 0.21 | 0.31 | 0.09 | 0.38 | 0.41 | 0.29 | 0.33 | 0.15 | 0.39 | 0.28 |

Table S6: Observed prediction accuracy (PH, Flint NAM families). Family of column k predicted with family of line j

|  |  | Predicted family | | | | | | | | | | |
| --- | --- | --- | --- | --- | --- | --- | --- | --- | --- | --- | --- | --- |
|  |  | D152 | EC49A | EZ5 | F03802 | F2 | F283 | F64 | UH006 | UH009 | DK105 | Average |
| Calibration  family | D152 | . | 0.37 | 0.09 | 0.24 | 0.48 | 0.55 | -0.20 | 0.43 | 0.48 | 0.50 | 0.33 |
|  | EC49A | 0.32 | . | 0.39 | -0.09 | -0.09 | 0.40 | 0.24 | 0.24 | 0.03 | 0.30 | 0.19 |
|  | EZ5 | 0.24 | 0.34 | . | 0.11 | 0.21 | 0.20 | 0.63 | 0.03 | -0.29 | 0.18 | 0.18 |
|  | F03802 | 0.37 | -0.23 | 0.38 | . | 0.48 | 0.20 | 0.04 | 0.54 | 0.05 | 0.17 | 0.22 |
|  | F2 | 0.55 | -0.04 | 0.30 | 0.34 | . | 0.39 | 0.21 | 0.37 | 0.12 | 0.26 | 0.28 |
|  | F283 | 0.41 | 0.51 | 0.13 | 0.24 | 0.37 | . | -0.03 | 0.67 | 0.21 | 0.67 | 0.35 |
|  | F64 | -0.11 | 0.28 | 0.83 | 0.08 | 0.11 | 0.03 | . | -0.07 | -0.35 | -0.16 | 0.07 |
|  | UH006 | 0.46 | 0.42 | 0.18 | 0.38 | 0.42 | 0.75 | -0.05 | . | 0.42 | 0.64 | 0.40 |
|  | UH009 | 0.43 | 0.05 | -0.36 | 0.03 | 0.11 | 0.30 | -0.36 | 0.40 | . | 0.29 | 0.10 |
|  | DK105 | 0.55 | 0.44 | 0.29 | 0.11 | 0.28 | 0.78 | -0.06 | 0.56 | 0.21 | . | 0.35 |
|  | Average | 0.36 | 0.24 | 0.25 | 0.16 | 0.26 | 0.40 | 0.05 | 0.35 | 0.10 | 0.32 | 0.25 |

Table S7: CDpop computed with the $\boldsymbol{\lambda}$ estimated by REML for DMY (Dent NAM families). The size of the families are indicated between brackets.

|  |  | Predicted family | | | | | | | | | | |
| --- | --- | --- | --- | --- | --- | --- | --- | --- | --- | --- | --- | --- |
|  |  | B73  (64) | D06  (99) | D09  (100) | EC169  (66) | F252  (96) | F618  (104) | Mo17  (53) | UH250  (94) | UH304  (81) | W117  (84) | **Average** |
| Calibration  family | B73 (64) | . | 0.35 | 0.32 | 0.48 | 0.31 | 0.35 | 0.34 | 0.37 | 0.30 | 0.32 | 0.35 |
|  | D06 (99) | 0.36 | . | 0.58 | 0.42 | 0.34 | 0.36 | 0.34 | 0.58 | 0.34 | 0.34 | 0.41 |
|  | D09 (100) | 0.36 | 0.64 | . | 0.38 | 0.38 | 0.36 | 0.35 | 0.56 | 0.39 | 0.34 | 0.42 |
|  | EC169 (66) | 0.48 | 0.41 | 0.34 | . | 0.31 | 0.35 | 0.35 | 0.39 | 0.30 | 0.32 | 0.36 |
|  | F252 (96) | 0.38 | 0.41 | 0.41 | 0.38 | . | 0.37 | 0.39 | 0.43 | 0.41 | 0.39 | 0.40 |
|  | F618 (104) | 0.31 | 0.32 | 0.28 | 0.31 | 0.27 | . | 0.28 | 0.32 | 0.28 | 0.28 | 0.29 |
|  | Mo17 (53) | 0.34 | 0.33 | 0.31 | 0.35 | 0.32 | 0.31 | . | 0.36 | 0.28 | 0.34 | 0.33 |
|  | UH250 (94) | 0.36 | 0.57 | 0.50 | 0.39 | 0.35 | 0.36 | 0.36 | . | 0.31 | 0.33 | 0.39 |
|  | UH304 (81) | 0.24 | 0.27 | 0.28 | 0.24 | 0.27 | 0.26 | 0.23 | 0.26 | . | 0.24 | 0.26 |
|  | W117 (84) | 0.31 | 0.32 | 0.29 | 0.31 | 0.31 | 0.31 | 0.32 | 0.31 | 0.28 | . | 0.31 |
|  | Average | 0.35 | 0.40 | 0.37 | 0.36 | 0.32 | 0.34 | 0.33 | 0.40 | 0.32 | 0.32 | 0.35 |

Table S8: CDpop computed with the $\boldsymbol{\lambda}$ estimated by REML for PH (Dent NAM families). The size of the families are indicated between brackets.

|  |  | Predicted family | | | | | | | | | | |
| --- | --- | --- | --- | --- | --- | --- | --- | --- | --- | --- | --- | --- |
|  |  | B73  (64) | D06  (99) | D09  (100) | EC169  (66) | F252  (96) | F618  (104) | Mo17  (53) | UH250  (94) | UH304  (81) | W117  (84) | **Average** |
| Calibration  family | B73 (64) | . | 0.39 | 0.36 | 0.53 | 0.35 | 0.39 | 0.38 | 0.41 | 0.33 | 0.36 | 0.39 |
|  | D06 (99) | 0.43 | . | 0.72 | 0.51 | 0.43 | 0.44 | 0.41 | 0.70 | 0.42 | 0.41 | 0.50 |
|  | D09 (100) | 0.39 | 0.70 | . | 0.41 | 0.41 | 0.40 | 0.38 | 0.61 | 0.44 | 0.38 | 0.46 |
|  | EC169 (66) | 0.54 | 0.47 | 0.39 | . | 0.36 | 0.40 | 0.40 | 0.45 | 0.34 | 0.37 | 0.41 |
|  | F252 (96) | 0.37 | 0.40 | 0.40 | 0.37 | . | 0.35 | 0.38 | 0.42 | 0.40 | 0.37 | 0.38 |
|  | F618 (104) | 0.44 | 0.45 | 0.42 | 0.44 | 0.39 | . | 0.40 | 0.45 | 0.40 | 0.41 | 0.42 |
|  | Mo17 (53) | 0.35 | 0.34 | 0.32 | 0.36 | 0.33 | 0.32 | . | 0.37 | 0.29 | 0.35 | 0.34 |
|  | UH250 (94) | 0.46 | 0.71 | 0.63 | 0.49 | 0.44 | 0.45 | 0.44 | . | 0.41 | 0.41 | 0.49 |
|  | UH304 (81) | 0.33 | 0.37 | 0.39 | 0.34 | 0.38 | 0.35 | 0.33 | 0.36 | . | 0.34 | 0.35 |
|  | W117 (84) | 0.39 | 0.40 | 0.38 | 0.39 | 0.39 | 0.39 | 0.41 | 0.39 | 0.37 | . | 0.39 |
|  | Average | 0.41 | 0.47 | 0.44 | 0.43 | 0.39 | 0.39 | 0.39 | 0.46 | 0.38 | 0.38 | 0.41 |

Table S9: CDpop computed with the $\boldsymbol{\lambda}$ estimated by REML for AD (Flint NAM families). The size of the families are indicated between brackets.

|  |  | Predicted family | | | | | | | | | | |
| --- | --- | --- | --- | --- | --- | --- | --- | --- | --- | --- | --- | --- |
|  |  | D152  (72) | EC49A  (29) | EZ5  (26) | F03802  (129) | F2  (54) | F283  (133) | F64  (64) | UH006  (94) | UH009  (98) | DK105  (95) | **Average** |
| **Calibration**  **family** | D152 (72) | . | 0.25 | 0.21 | 0.25 | 0.27 | 0.26 | 0.22 | 0.25 | 0.23 | 0.28 | 0.25 |
|  | EC49A (29) | 0.32 | . | 0.36 | 0.29 | 0.33 | 0.34 | 0.33 | 0.29 | 0.28 | 0.32 | 0.32 |
|  | EZ5 (26) | 0.26 | 0.34 | . | 0.31 | 0.32 | 0.30 | 0.36 | 0.27 | 0.30 | 0.25 | 0.30 |
|  | F03802 (129) | 0.36 | 0.33 | 0.34 | . | 0.35 | 0.53 | 0.35 | 0.31 | 0.30 | 0.41 | 0.37 |
|  | F2 (54) | 0.28 | 0.27 | 0.28 | 0.25 | . | 0.28 | 0.25 | 0.34 | 0.22 | 0.23 | 0.27 |
|  | F283 (133) | 0.40 | 0.40 | 0.38 | 0.55 | 0.41 | . | 0.37 | 0.41 | 0.40 | 0.51 | 0.43 |
|  | F64 (64) | 0.33 | 0.39 | 0.45 | 0.38 | 0.36 | 0.37 | . | 0.32 | 0.37 | 0.34 | 0.37 |
|  | UH006 (94) | 0.32 | 0.28 | 0.28 | 0.25 | 0.40 | 0.33 | 0.26 | . | 0.39 | 0.30 | 0.31 |
|  | UH009 (98) | 0.27 | 0.25 | 0.28 | 0.25 | 0.26 | 0.30 | 0.28 | 0.38 | . | 0.28 | 0.28 |
|  | DK105 (95) | 0.45 | 0.38 | 0.31 | 0.42 | 0.34 | 0.51 | 0.35 | 0.38 | 0.37 | . | 0.39 |
|  | Average | 0.33 | 0.32 | 0.32 | 0.33 | 0.34 | 0.36 | 0.31 | 0.33 | 0.32 | 0.32 | 0.33 |

Table S10: CDpop computed with the $\boldsymbol{\lambda}$ estimated by REML for DMY (Flint NAM families). The size of the families are indicated between brackets.

|  |  | Predicted family | | | | | | | | | | |
| --- | --- | --- | --- | --- | --- | --- | --- | --- | --- | --- | --- | --- |
|  |  | D152  (72) | EC49A  (29) | EZ5  (26) | F03802  (129) | F2  (54) | F283  (133) | F64  (64) | UH006  (94) | UH009  (98) | DK105  (95) | **Average** |
| **Calibration**  **family** | D152 (72) | . | 0.28 | 0.25 | 0.28 | 0.31 | 0.30 | 0.25 | 0.29 | 0.26 | 0.32 | 0.28 |
|  | EC49A (29) | 0.12 | . | 0.13 | 0.11 | 0.12 | 0.13 | 0.12 | 0.10 | 0.10 | 0.12 | 0.12 |
|  | EZ5 (26) | 0.17 | 0.22 | . | 0.20 | 0.21 | 0.19 | 0.24 | 0.17 | 0.20 | 0.16 | 0.20 |
|  | F03802 (129) | 0.29 | 0.26 | 0.28 | . | 0.27 | 0.43 | 0.29 | 0.23 | 0.23 | 0.32 | 0.29 |
|  | F2 (54) | 0.29 | 0.27 | 0.29 | 0.26 | . | 0.29 | 0.25 | 0.35 | 0.23 | 0.24 | 0.27 |
|  | F283 (133) | 0.38 | 0.39 | 0.36 | 0.53 | 0.39 | . | 0.35 | 0.39 | 0.38 | 0.49 | 0.41 |
|  | F64 (64) | 0.25 | 0.30 | 0.34 | 0.29 | 0.27 | 0.28 | . | 0.24 | 0.29 | 0.26 | 0.28 |
|  | UH006 (94) | 0.31 | 0.27 | 0.27 | 0.25 | 0.40 | 0.32 | 0.25 | . | 0.38 | 0.29 | 0.31 |
|  | UH009 (98) | 0.19 | 0.18 | 0.21 | 0.18 | 0.18 | 0.22 | 0.21 | 0.27 | . | 0.20 | 0.20 |
|  | DK105 (95) | 0.40 | 0.34 | 0.28 | 0.38 | 0.31 | 0.46 | 0.31 | 0.33 | 0.33 | . | 0.35 |
|  | Average | 0.27 | 0.28 | 0.27 | 0.28 | 0.27 | 0.29 | 0.25 | 0.26 | 0.27 | 0.27 | 0.27 |

Table S11: CDpop computed with the $\boldsymbol{\lambda}$ estimated by REML for PH (Flint NAM families). The size of the families are indicated between brackets.

|  |  | Predicted family | | | | | | | | | | |
| --- | --- | --- | --- | --- | --- | --- | --- | --- | --- | --- | --- | --- |
|  |  | D152  (72) | EC49A  (29) | EZ5  (26) | F03802  (129) | F2  (54) | F283  (133) | F64  (64) | UH006  (94) | UH009  (98) | DK105  (95) | **Average** |
| **Calibration**  **family** | D152 (72) | . | 0.29 | 0.26 | 0.30 | 0.33 | 0.32 | 0.26 | 0.31 | 0.28 | 0.34 | 0.30 |
|  | EC49A (29) | 0.20 | . | 0.23 | 0.19 | 0.21 | 0.22 | 0.21 | 0.18 | 0.18 | 0.21 | 0.20 |
|  | EZ5 (26) | 0.15 | 0.20 | . | 0.18 | 0.19 | 0.17 | 0.21 | 0.16 | 0.17 | 0.14 | 0.18 |
|  | F03802 (129) | 0.29 | 0.27 | 0.28 | . | 0.28 | 0.43 | 0.29 | 0.23 | 0.24 | 0.33 | 0.29 |
|  | F2 (54) | 0.24 | 0.23 | 0.24 | 0.21 | . | 0.24 | 0.21 | 0.29 | 0.19 | 0.20 | 0.23 |
|  | F283 (133) | 0.39 | 0.39 | 0.37 | 0.54 | 0.40 | . | 0.36 | 0.40 | 0.39 | 0.49 | 0.41 |
|  | F64 (64) | 0.28 | 0.33 | 0.38 | 0.32 | 0.30 | 0.31 | . | 0.27 | 0.32 | 0.29 | 0.31 |
|  | UH006 (94) | 0.34 | 0.29 | 0.29 | 0.27 | 0.43 | 0.35 | 0.27 | . | 0.41 | 0.32 | 0.33 |
|  | UH009 (98) | 0.24 | 0.23 | 0.25 | 0.22 | 0.23 | 0.27 | 0.26 | 0.34 | . | 0.25 | 0.26 |
|  | DK105 (95) | 0.43 | 0.37 | 0.30 | 0.41 | 0.33 | 0.49 | 0.34 | 0.37 | 0.36 | . | 0.38 |
|  | Average | 0.28 | 0.29 | 0.29 | 0.29 | 0.30 | 0.31 | 0.27 | 0.28 | 0.28 | 0.28 | 0.29 |

Table S12: Correlation between expected (CDpop) and observed accuracy in scenario S1 (Dent). The size of the families are indicated between brackets.

|  | Predicted family | | | | | | | | | |  |
| --- | --- | --- | --- | --- | --- | --- | --- | --- | --- | --- | --- |
|  | B73  (64) | D06  (99) | D09  (100) | EC169  (66) | F252  (96) | F618  (104) | Mo17  (53) | UH250  (94) | UH304  (81) | W117  (84) | **Average** |
| AD | 0.68 | 0.85 | 0.76 | -0.94 | 0.78 | 0.26 | 0.29 | 0.76 | 0.04 | 0.88 | 0.44 |
| DMY | 0.53 | 0.61 | 0.59 | 0.75 | 0.28 | 0.81 | 0.03 | 0.64 | 0.00 | 0.73 | 0.50 |
| PH | -0.02 | 0.85 | 0.82 | 0.5 | 0.38 | 0.1 | 0.36 | 0.57 | 0.33 | 0.31 | 0.42 |
|  |  |  |  |  |  |  |  |  |  |  |  |

Table S13: Correlation between expected (CDpop) and observed accuracy in scenario S1 (Flint). The size of the families are indicated between brackets.

|  | Predicted family | | | | | | | | | |  |
| --- | --- | --- | --- | --- | --- | --- | --- | --- | --- | --- | --- |
|  | D152  (72) | EC49A  (29) | EZ5  (26) | F03802  (129) | F2  (54) | F283  (133) | F64  (64) | UH006  (94) | UH009  (98) | DK105  (95) | **Average** |
| AD | 0.08 | 0.28 | 0.64 | 0.37 | 0.72 | 0.37 | 0.68 | 0.63 | -0.03 | 0.51 | 0.42 |
| DMY | 0.33 | 0.73 | 0.46 | 0.78 | 0.87 | 0.24 | 0.02 | 0.93 | 0.79 | 0.51 | 0.57 |
| PH | 0.35 | 0.57 | 0.44 | 0.24 | 0.63 | 0.44 | -0.50 | 0.68 | 0.45 | 0.53 | 0.38 |
|  |  |  |  |  |  |  |  |  |  |  |  |

Table S14: Observed and expected (CDpop) accuracies for Scenario 3 when each NAM family is predicted by the corresponding panel or the whole NAM population is predicted by the panel. Correlation between predicted and observed accuracies are given considering or not (between parentheses) results obtained for the prediction of the whole NAM population, Dent NAM families. The size of the families are indicated between brackets.

|  |  | | Predicted family | | | | | | | | |  | Average  Within NAM | Whole NAM | Correlation |
| --- | --- | --- | --- | --- | --- | --- | --- | --- | --- | --- | --- | --- | --- | --- | --- |
|  | | | B73  (64) | D06  (99) | D09  (100) | EC169  (66) | F252  (96) | F618  (104) | Mo17  (53) | UH250  (94) | UH304  (81) | W117  (84) |  |  |  |
| Accuracy AD | | | -0.16 | 0.34 | 0.43 | -0.01 | 0.39 | 0.34 | 0.41 | 0.31 | -0.14 | 0.54 | 0.24 | 0.67 | 0.25 (-0.27) |
| CDPop AD | | | 0.47 | 0.48 | 0.43 | 0.46 | 0.40 | 0.40 | 0.43 | 0.47 | 0.39 | 0.39 | 0.43 | 0.60^a^ |  |
| Accuracy DMY | | | -0.20 | 0.15 | 0.34 | -0.04 | 0.30 | -0.23 | -0.13 | 0.29 | 0.16 | -0.01 | 0.06 | 0.43 | 0.39 (-0.01) |
| CDPop DMY | | | 0.46 | 0.46 | 0.42 | 0.44 | 0.38 | 0.38 | 0.41 | 0.45 | 0.37 | 0.37 | 0.41 | 0.59 |  |
| Accuracy PH | | | 0.01 | 0.28 | 0.42 | 0.23 | 0.19 | -0.06 | 0.01 | 0.30 | 0.10 | 0.33 | 0.18 | 0.43 | 0.45 (0.17) |
| CDPop PH | | | 0.43 | 0.43 | 0.39 | 0.41 | 0.35 | 0.35 | 0.38 | 0.42 | 0.34 | 0.35 | 0.39 | 0.56 |  |
| Mean Accuracy | | | -0.11 | 0.25 | 0.40 | 0.06 | 0.29 | 0.01 | 0.09 | 0.30 | 0.04 | 0.29 | 0.16 | 0.51 |  |
| Mean CD | | | 0.45 | 0.46 | 0.41 | 0.44 | 0.38 | 0.38 | 0.41 | 0.45 | 0.37 | 0.37 | 0.41 | 0.58 |  |
|  | |  |  |  |  |  |  |  |  |  |  |  |  |  |  |

Table S15: Observed and expected (CDpop) accuracies for Scenario 3 when each NAM family is predicted by the corresponding panel or the whole NAM population is predicted by the panel. Correlation between predicted and observed accuracies are given considering or not (between parentheses) results obtained for the prediction of the whole NAM population, Flint NAM families. The size of the families are indicated between brackets.

|  |  | | Predicted family | | | | | | | | |  | Average  Within NAM | Whole NAM | Correlation |
| --- | --- | --- | --- | --- | --- | --- | --- | --- | --- | --- | --- | --- | --- | --- | --- |
|  | | | D152  (72) | EC49A  (29) | EZ5  (26) | F03802  (129) | F2  (54) | F283  (133) | F64  (64) | UH006  (94) | UH009  (98) | DK105  (95) |  |  |  |
| Accuracy AD | | | 0.21 | 0.39 | 0.69 | 0.27 | 0.18 | 0.29 | 0.52 | 0.45 | 0.43 | 0.69 | 0.41 | 0.72 | 0.21 (-0.31) |
| CDPop AD | | | 0.51 | 0.47 | 0.43 | 0.52 | 0.53 | 0.56 | 0.49 | 0.56 | 0.53 | 0.55 | 0.51 | 0.68 |  |
| Accuracy DMY | | | -0.08 | 0.44 | 0.27 | 0.14 | 0.58 | 0.41 | 0.14 | 0.27 | -0.06 | 0.32 | 0.24 | 0.32 | 0.12 (0.03) |
| CDPop DMY | | | 0.43 | 0.39 | 0.37 | 0.43 | 0.44 | 0.47 | 0.42 | 0.45 | 0.43 | 0.45 | 0.43 | 0.45 |  |
| Accuracy PH | | | 0.34 | 0.37 | 0.58 | 0.22 | 0.31 | 0.50 | 0.61 | 0.39 | 0.37 | 0.42 | 0.41 | 0.42 | 0.20 (-0.32) |
| CDPop PH | | | 0.47 | 0.44 | 0.41 | 0.48 | 0.49 | 0.52 | 0.46 | 0.51 | 0.49 | 0.51 | 0.48 | 0.51 |  |
| Mean Accuracy | | | 0.16 | 0.40 | 0.52 | 0.21 | 0.36 | 0.40 | 0.42 | 0.37 | 0.25 | 0.48 | 0.35 | 0.54 |  |
| Mean CD | | | 0.47 | 0.43 | 0.40 | 0.48 | 0.48 | 0.52 | 0.46 | 0.51 | 0.48 | 0.50 | 0.47 | 0.63 |  |
|  | |  |  |  |  |  |  |  |  |  |  |  |  |  |  |


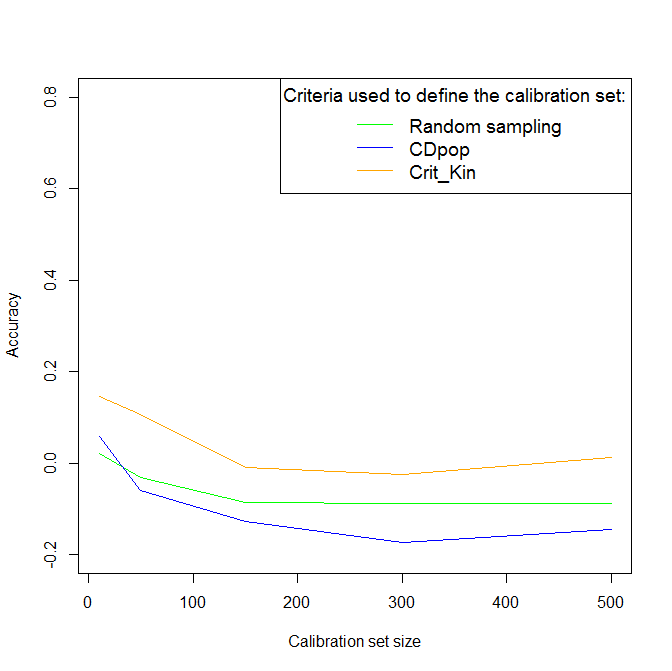


Figure S1 : Observed prediction accuracies obtained in scenario OT1 for family UH304 for trait DMY.


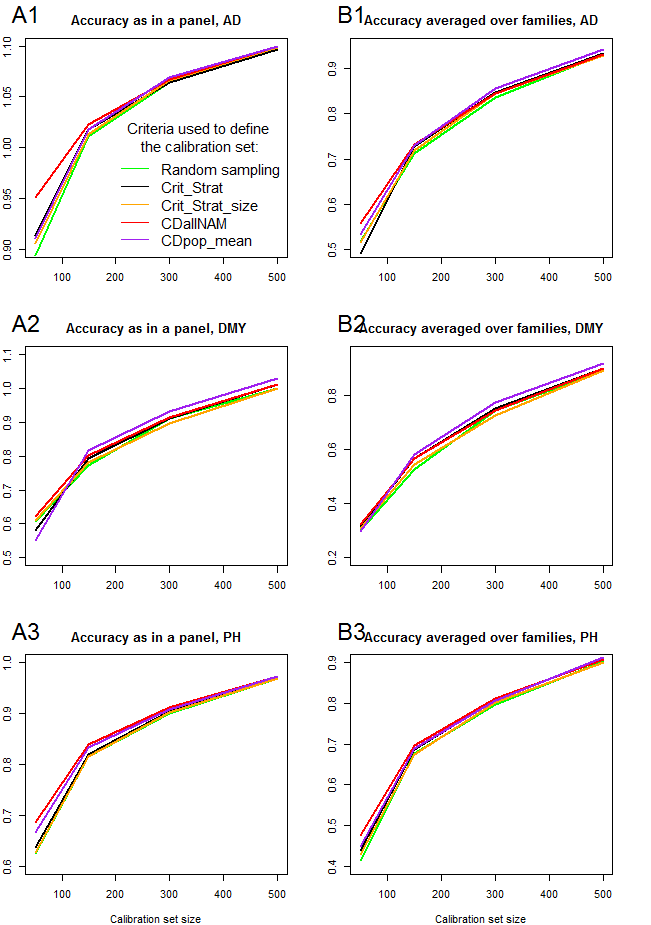

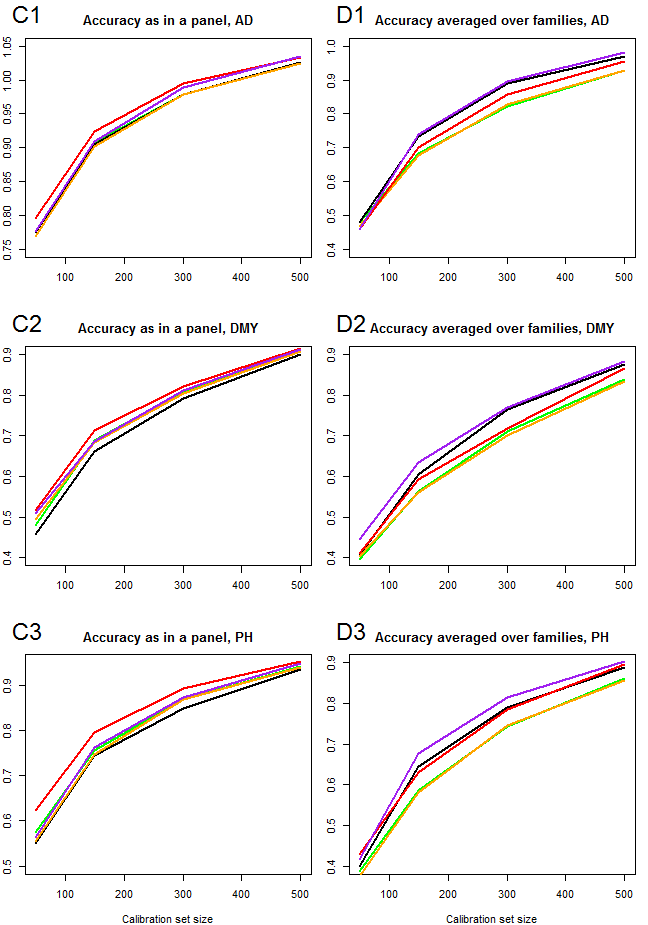


Figure S2 : Observed accuracies obtained in scenario OT3 for the Dent (A and B) and the Flint NAM (C and D). In OT3 the calibration set is sampled among all families to predict simultaneously all families as if it was a single unstructured population. Accuracies are then computed for intra-family predictions (B and D) and for global predictions as if it was a single unstructured population (A and C).


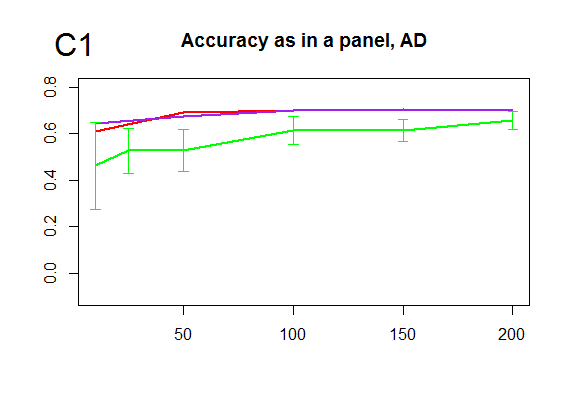

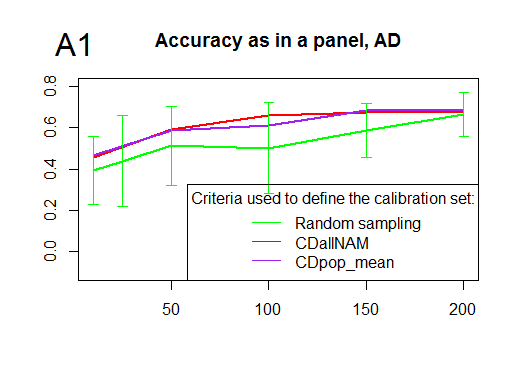

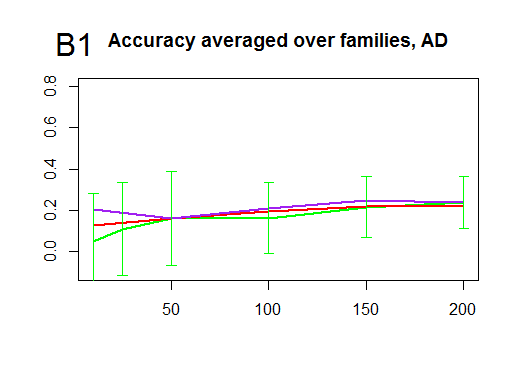

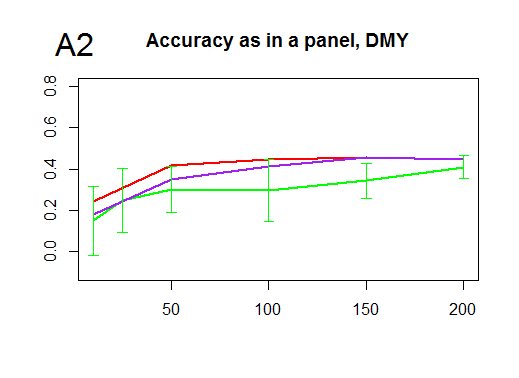

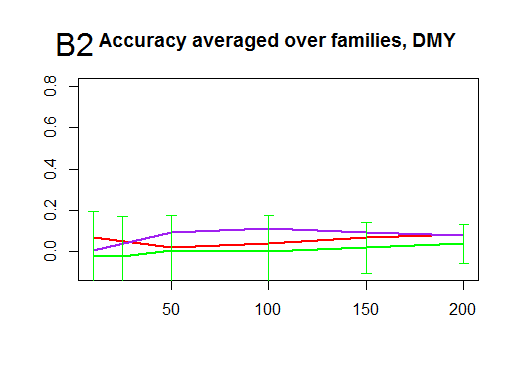

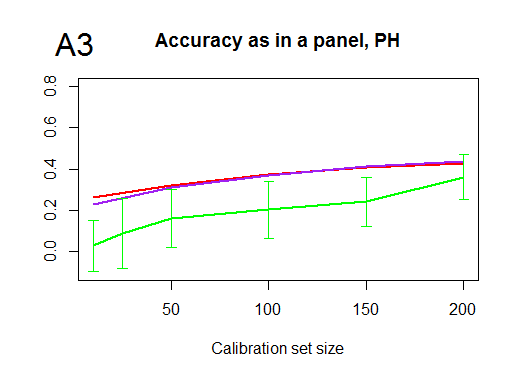

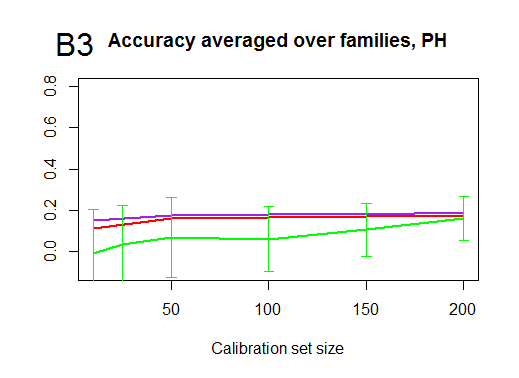

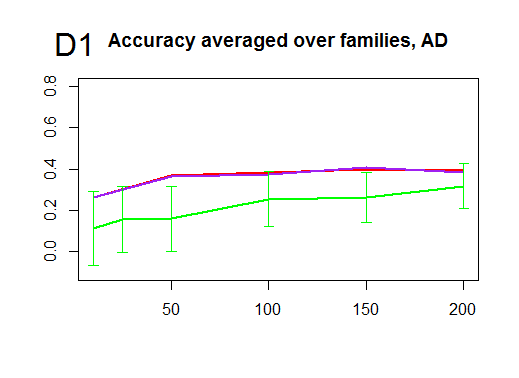

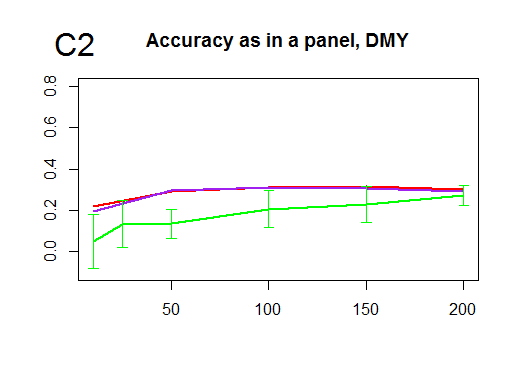

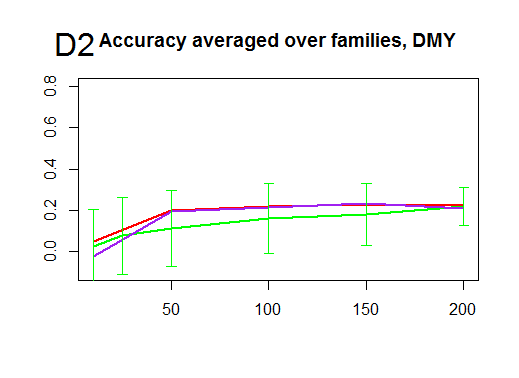

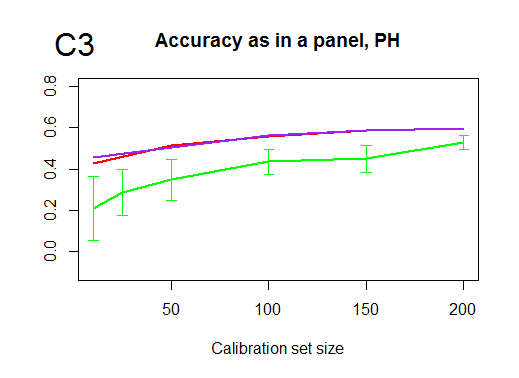

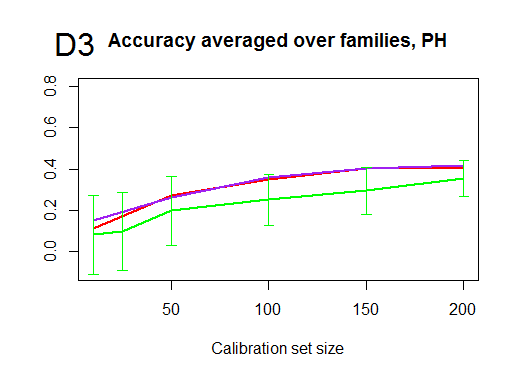


Figure S3: Observed accuracies obtained in scenario OT4 for the Dent (A and B) and the Flint NAM (C and D). In OT4 the calibration set is sampled in the highly diverse panel to predict all NAM families simultaneously. Accuracies are then computed for intra-family predictions (A and C) and for global predictions as if it was an unstructured population (B and D).


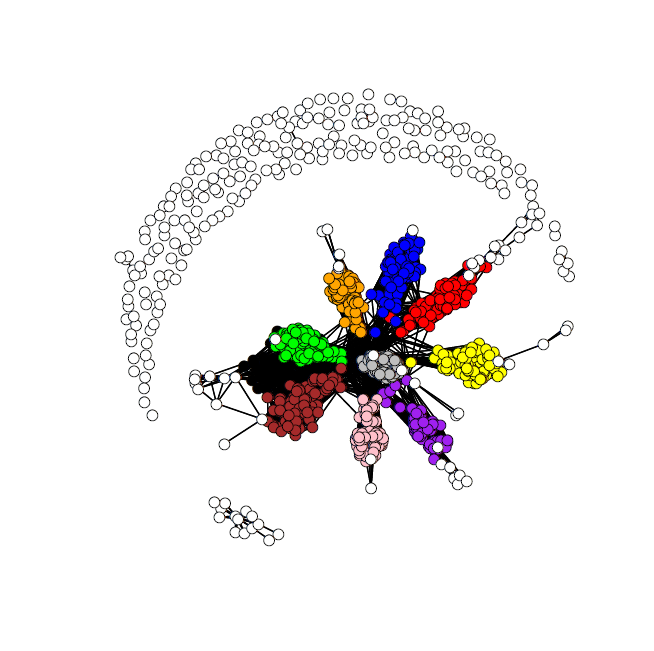


B73

D06

D09

EC169

F252

F618

Mo17

UH250

UH304

W117

F353


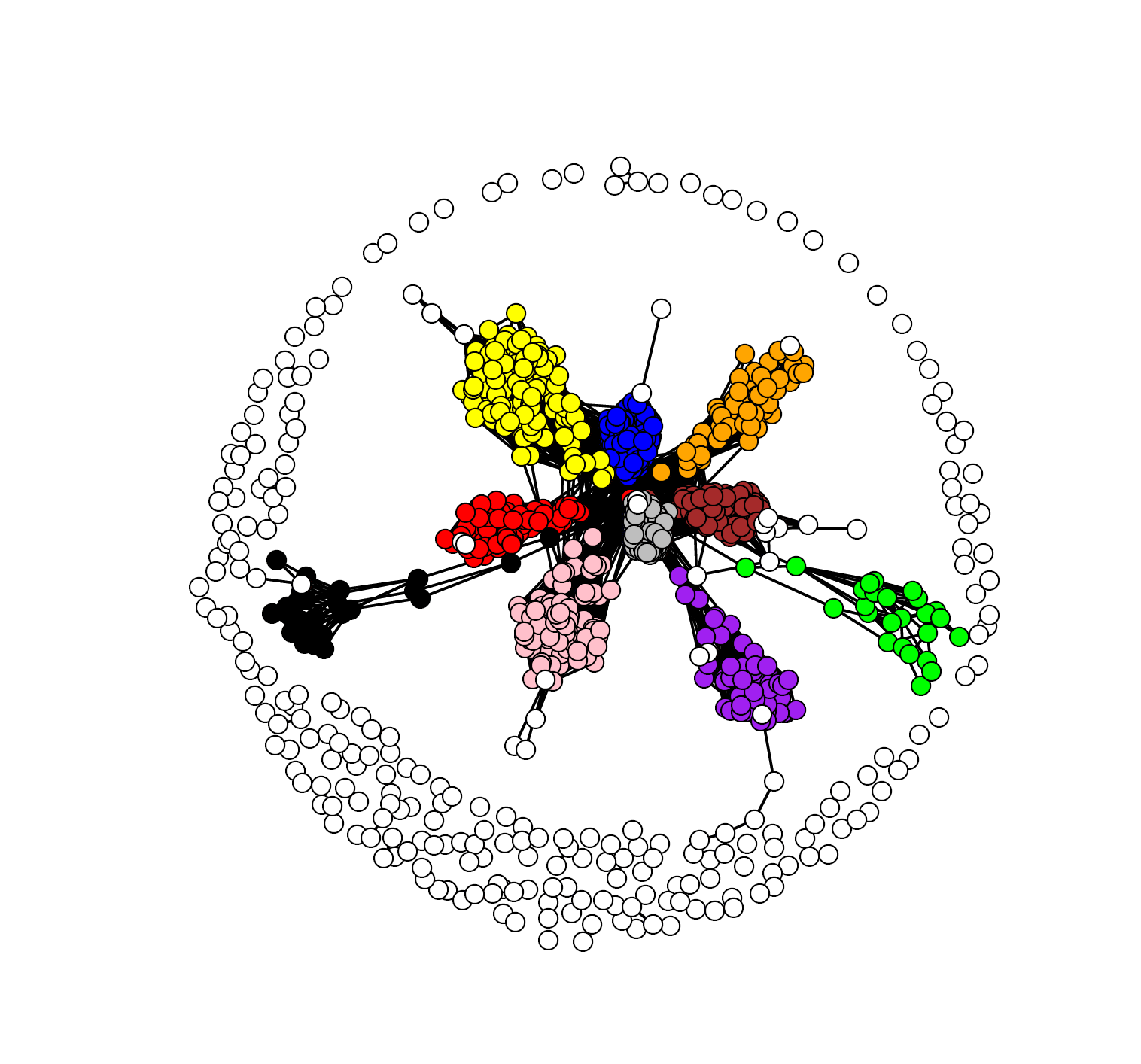


F64

F2

F283

EZ5

UH006

DK105

UH009

D152

EC49A

F03802

UH005

Flint

Dent

Figure S4 : Kinship networks for the dent (left) and flint (right) populations. In each network, individuals are plotted as white dots for the panel lines and as colored dots for NAM lines. Each color corresponds to a different NAM family. Dots connected with a line are related by a kinship coefficient above 0.5. It can be noted that NAM individuals from a given family are grouped together and positioned between their two parental lines: the “central” line of the NAM design (F353 for the dent lines and UH005 for the flint lines) and the alternative parent (B73, D06, D09, EC169, F252, F618, Mo17, UH250, UH304, W117, for the dent parents, D152, EC149A, F03802, F2, F283, F64, UH006, UH009, DK105, for the flint parents). All parents but EZ5 belong to the panels.

##################################################################################################################################

# Optimization of training set for genomic selection in highly structured material (multi-familial design)

# In this script you will find an example on how to optimize a calibration set to maximize prediction accuracy within a particular family

# using the criterion CDpop. The calibration set is sampled among all the families except one which is the family you want to predict.

# All details can be found in Rincent et al. 2017 TAG (Predicting genomic selection efficiency to optimize calibration set and to assess

# prediction accuracy in highly structured populations)

# renaud.rincent@inra.fr 27/06/2017

##################################################################################################################################

rm(list=ls())

##############################################################

# Load data

##############################################################

matA=read.table(...) # kinship matrix between all individuals

matA=as.matrix(matA)

invA=solve(matA) # Invert the kinship matrix

pop2=read.table(...) # pop2 is a numeric vector indicating to which family belong each individual

# The individuals should be ranked in the same order in matA and pop2

##############################################################

# Sampling optimized calibration set to predict a particular family using CDpop

##############################################################

# I/ Initialization

##############################################################

h2=0.5 # Set a heritability similar to the heritabilities of the traits of interest (or choose an intermediate value of 0.5)

lambda=1/h2-1

Nind_in_Sample=50 # Size of the calibration set

popPred=1 # popPred is the family you want to predict (reference to vector pop2)

nIter=3000 # Number of iterations for the exchange algorithm (make sure it is sufficient by checking that CDpop # reach a plateau)

CDpopExchange=rep(NA,nIter)

Fam=which(pop2==popPred) # individuals in the test set (the family you want to predict)

T=matrix(0,Nind_in_Sample+length(Fam),length(Fam)) # T is the matrix of contrasts, each column is a contrast

T[(Nind_in_Sample+1):nrow(T),]=-1/length(Fam) # between one individual of the test set (the predicted

for (i in 1:length(Fam)) { T[(Nind_in_Sample+i),i]=1-1/length(Fam) } # family) and the mean of its family

X<-rep(1,Nind_in_Sample) # Design matrixes

Ident<-diag(Nind_in_Sample)

M<-Ident-(X%*%solve(t(X)%*%X) %*% t(X) )

NotSampled1=which(pop2!=popPred) # individuals which can potentialy be in the calibration set

Sample1<-sample(NotSampled1,Nind_in_Sample) # Initial calibration set

SaveSample=Sample1

NotSampled<-NotSampled1[-match(Sample1,NotSampled1)]

Z=matrix(0,Nind_in_Sample,Nind_in_Sample+length(Fam)) # Design matrix indicating which individuals are in the calibration set

for (i in 1:Nind_in_Sample) { Z[i,i]=1 }

indi=c(Sample1,Fam) # individuals in the calibration or in the predicted set

matA1=matA[indi,indi] # kinship between these individuals

invA1=solve(matA1)

matCD<-(t(T)%*%(matA1-lambda*solve(t(Z)%*%M%*%Z + lambda*invA1))%*%T)/(t(T)%*%matA1%*%T) # compute the CDs of the contrasts

CD=diag(matCD)

CDpopSave=mean(CD) # Value of CDpop for this calibration set

CDpopExchange[1]=CDpopSave

# II/ Exchange algorithm

##############################################################

# In the exchange algorithm, at each iteration one individual in the calibration set is exchanged

# with an individual that was not in the calibration set. The CDpop of the new calibration set

# is computed, and the exchange is accepted is CDpop increased, rejected otherwise.

cpt2=1

while (cpt2<nIter) {

cpt2=cpt2+1

NotSampled=NotSampled1[-match(Sample1,NotSampled1)] # Individuals not in the calibration set

# Remove one individual (randomly choosen) from the calibration set

Sample2=sample(Sample1,1)

# Select one individual (randomly choosen) from the individuals that are not in the calibration set

Sample3=sample(NotSampled,1)

# New calibration set

Sample4=c(Sample3,Sample1[Sample1!=Sample2])

NotSampled=NotSampled1[-Sample4] # Individuals that are not in the new calibration set

# Calculate the CDpop of this new calibration set

indi=c(Sample4,Fam)

matA1=matA[indi,indi]

invA1=solve(matA1)

matCD<-(t(T)%*%(matA1-lambda*solve(t(Z)%*%M%*%Z + lambda*invA1))%*%T)/(t(T)%*%matA1%*%T)

CD=diag(matCD)

if (mean(CD)>CDpopSave) { Sample1=Sample4

CDpopSave=mean(CD)

}

CDpopExchange[cpt2]=CDpopSave

}

SampleOptimiz=Sample1 # Optimized calibration set

plot(CDpopExchange) # Values of CDpop taken during the exchange algorithm, make sure that you took sufficient iterations

# by checking that you reach a plateau. Also make sure that you are not stuck to a local

# optimum by runing the exchange algorithms few times with different initial calibration sets

# End of script

############################################################################################################################
